# Feasibility and acceptability of electronic EQ-5D-5L for routine measurement of HRQOL in patients with chronic musculoskeletal problems in Hong Kong primary care

**DOI:** 10.1186/s12955-022-02047-0

**Published:** 2022-09-20

**Authors:** Amy Pui Pui Ng, Kiki Sze Nga Liu, Will Ho Gi Cheng, Carlos King Ho Wong, John King Yiu Cheng, Joyce Sau Mei Lam, Calvin Kalun Or, Emily Tsui Yee Tse, Cindy Lo Kuen Lam

**Affiliations:** 1 Department of Family Medicine and Primary Care, The University of Hong Kong-Shenzhen Hospital, 1 Haiyuan 1st Rd, Futian District, Shenzhen, 518009 Guangdong Province China; 2 Department of Family Medicine and Primary Care, School of Clinical Medicine, Li Ka Shing Faculty of Medicine, The University of Hong Kong, 3rd Floor, Ap Lei Chau Clinic, 161 Main Street, Ap Lei Chau, Hong Kong SAR China; 3 Department of Pharmacology and Pharmacy, Li Ka Shing Faculty of Medicine, The University of Hong Kong, Pok Fu Lam, Hong Kong SAR China; 4 Laboratory of Data Discovery for Health (D²4H), Hong Kong Science and Technology Park, Hong Kong SAR Sha Tin, China; 5 Department of Industrial and Manufacturing Systems Engineering, Faculty of Engineering, The University of Hong Kong, Pok Fu Lam, Hong Kong SAR China

**Keywords:** Health-related quality of life, Musculoskeletal problem, Electronic EQ-5D-5L, Feasibility, Acceptability

## Abstract

**Background:**

Information on HRQOL can enhance patient diagnosis and management but it is rarely available in routine clinical practice. This mixed-method study evaluated the feasibility and acceptability of the electronic EQ-5D-5L measurement of HRQOL in patients with chronic musculoskeletal problems in primary care.

**Methods:**

In three primary care clinics, 665 patients with musculoskeletal problems completed the electronic EQ-5D-5L and Visual Analogue Scale (e-EQ-5D-5L/VAS), and a questionnaire on socio-demographics, perceived ease of use (PEOU), and perceived usefulness (PU) at baseline and two follow-ups. Patient completion and response rates, and time to complete the e-EQ-5D-5L/VAS were measured. During the same consultations, 49 doctors reviewed the e-EQ-5D-5L/VAS reports and completed a clinician questionnaire on PEOU, PU, and time spent to address each report. Individual interviews along with focus group discussions were conducted on patients, doctors, and research assistants for further exploration.

**Results:**

Mean completion time reduced from baseline to first and second follow-up (120.66, 83.99, and 105.22 s, respectively). Completion and response rates were high at each follow-up visit (> 99.8% and > 91.11%, respectively). Doctors needed less than 2 min to read the report but felt the time required to address the report was a significant barrier. Some patients had difficulties using e-platforms, in understanding or answering questions; but, PEOU improved with time (*p* < 0.001). Most patients found the e-platforms useful (> 85.3%). Clinicians agreed a great majority of the reports were easy to use (76.0–85.1%) and useful (69.2–72.0%), particularly aiding with a holistic view of the patient's musculoskeletal problem.

**Conclusion:**

The e-EQ-5D-5L/VAS is a feasible and acceptable measurement of HRQOL of patients with chronic musculoskeletal problems in routine primary care in Hong Kong which can assist real-time management decisions.

*Trial registration*: NCT03609762.

**Supplementary Information:**

The online version contains supplementary material available at 10.1186/s12955-022-02047-0.

## Background

Health-related quality of life (HRQOL) is a common patient-reported outcome (PRO) assessing a person’s subjective judgment on how their health impacts them physiologically and psychologically [1]. Increasingly, research shows that using PRO in clinical practice can assist joint decision-making in the diagnostic and management processes [2] and benefit patient care [3–7]. However, incorporating these measurements into routine clinical practice comes with challenges in terms of the feasibility and users’ perceptions of the measurements [7]. Increased workload [8–10] and a limited time to collect, analyze and interpret data [11] have led to calls for more efficient methods in obtaining data in routine clinical practice [10, 12], such as via electronic data collection and reporting [10]. Apart from reducing workload, clinicians can have immediate access to the results and track HRQOL changes [3, 8].

The EQ-5D-5L is a popular HRQOL measure [13] that assesses the patients’ self-perceived health-related barriers in daily function, pain, and psychological distress with five items. It was originally developed by the EuroQol Group and has been adapted to many other populations including the Chinese population in Hong Kong [13–15]. Its greatest advantage is that it is short, easy-to-complete and provides information on both the HRQOL profile and health utility of the patient [13]. An electronic version (e-EQ-5D-5L) is available and has been used in many HRQOL outcome studies [16–19]. Our previous study confirmed the validity, reliability and responsiveness of the e-EQ-5D-5L in Chinese primary care patients in Hong Kong [20]. Research supports that e-EQ-5D-5L measures in clinical practice identify more HRQOL problems and result in more actions by clinicians to tackle them [21]. It also has the potential to track the changes in HRQOL of an individual patient in clinical practice.

However, the implementation of electronic health information systems can bring with it challenges related to feasibility and users’ acceptance of the technology [22]. Feasibility is concerned with the difficulty of applying instruments in a population [23]. Existing EQ-5D studies have used missing values, completion rates and time, response rates, and qualitative statements about completion as indicators of feasibility [23]. Past studies on EQ-5D [23, 24] and other electronic PRO measures [25, 26] cited satisfactory completion rates from 80 to 95%. Studies on EQ-5D response rates have shown inconsistent results with a Swedish study of arthroplasty patients showing that response rate to a web-based version (49%) was worse than that to a paper-based version (92%) [27], whereas a study on English residents showed a better response rate of 73% using a mobile-version than that (66%) of using paper-version [28]. However, the differences could be due to age, education levels, and health status. Acceptability is a multifaceted concept that represents the degree to which those providing or receiving a healthcare intervention consider it to be suitable, based on expected or actual cognitive and emotional responses to the intervention. It consists of seven component constructs: affective attitude, burden, perceived effectiveness, ethicality, intervention coherence, opportunity costs, and self-efficacy [29]. Earlier studies on acceptability of electronic HRQOL measures used different indictors of overall satisfaction, willingness to use and perceived usefulness, resulting in a wide variation in rates ranging from 66 to 83% [30, 31].

Users’ negative perceptions of a technology can hamper its acceptability and implementation. In 1989 and years afterwards, Davis and colleagues introduced the Technology Acceptance Model (TAM) which offers an approach for analyzing individuals’ perceptions of a new technology [32–34], which can be used as more standardized indictors of acceptability. The model essentially specifies two beliefs: perceived usefulness (PU), the feeling that technology would lead to improvement in task performance, and perceived ease of use (PEOU), the feeling that using the technology is effort-free. The model was empirically tested, and PU and PEOU were shown to be crucial considerations in technology acceptance and implementation [33–35]. The model has been applied to studies that assessed information technology in healthcare [36, 37], which showed encouraging results on acceptance of electronic HQOOL measures [38, 39].

To our knowledge, there has not been a study assessing the feasibility and acceptability of the e-EQ-5D-5L among Chinese patients in routine primary care. Electronic administration can be challenging for older patients who have low education levels [40] and are not familiar with computer technology [41]. The Chinese (Hong Kong) e-EQ-5D-5L is a valid, reliable, sensitive, and responsive measurement of HRQOL of Chinese patients with musculoskeletal problems in routine clinical practice [20]. This study aimed to evaluate the feasibility and acceptability of an electronic platform for timely, regular measurement and reporting of e-EQ-5D-5L HRQOL data in assessing patients with chronic knee and back (collectively referred to as musculoskeletal) problems in primary care. We hypothesized that doctors and patients would find the electronic platform feasible with a completion rate of 90% or above [23] and acceptable with perceived usefulness of 80% or above [30], and that feasibility and acceptability might improve with repeated use.

## Methods

### Study design

This was a mixed-method study. The first part was a prospective longitudinal cohort study where quantitative data from patients and doctors were collected at baseline and two follow-ups. The second part involved qualitative interviews of the patients, doctors, and research assistants (RAs) to further explore feasibility and acceptability. The study was approved by the Institutional Review Boards (IRB) of the HKU/Hospital Authority West Cluster (reference number: UW 18–270) and the Hospital Authority Kowloon Central/ Kowloon East Clusters (reference number: KC/KE-20-0070/ER-1),

### Prospective longitudinal cohort study

#### Patient recruitment

This study is part of a larger single-blind cluster randomized controlled trial (RCT) to evaluate the effectiveness of measuring and reporting HRQOL in routine clinical practice and the protocol has been published [20]. All doctors from three public primary care clinics that implement routine measurement of HRQOL by an e-EQ-5D-5L were invited to participate and signed a consent form. Doctors were trained to interpret the e-EQ-5D-5L profile and visual analogue scale (VAS) scores (collectively referred to as e-EQ-5D-5L/VAS). All adults aged 18 years or above with symptomatic chronic knee and/or back problems visiting the clinics were invited to participate based on the RCT’s inclusion and exclusion criteria [20]. In brief, the inclusion criteria were adults aged 18 years or above; had a doctor-diagnosed symptomatic knee and/or back problem that was expected to last for one month or more; scheduled for at least one follow-up visit in the clinic within 12 months; had given written consent to participate in the study. The exclusion criteria were patients who had life expectancy less than 12 months (judged by the doctor), had current cancers undergoing active or palliative treatment, were too ill (physically or cognitively) to complete a questionnaire; were unable to communicate in Chinese; or did not give consent to participate in the study. All participating patients signed a written consent form. Each patient was assigned a unique QR code for access to the e-EQ-5D-5L survey platform to complete the Chinese e-EQ-5D-5L and EQ-VAS online with an iPad that was connected to a central server via the clinic public Wi-Fi. One item was presented per screen and the participant could choose to move to the next item after completion or to skip the item. The original 200 mm EQ-VAS was modified to 100 mm to fit into the computer screen. The detailed administration method of the e-EQ-5D-5L with screenshots is shown in the Additional file 1: Appendix 1. Trained research assistants (RAs) provided technical assistance and read out the questions to the respondents, if necessary. Upon completion, the report summarizing the EQ-5D dimensions, utility, and VAS scores was printed (Additional file 1: Appendix 2). Patients were given the report and passed it to their doctor during the consultation and were managed accordingly, then the doctor filled out information on the clinician report form regarding the patient’s condition and management, and a questionnaire on the doctor’s PEOU and PU of the EQ-5D-5L/VAS report. After that, patients completed an e-questionnaire on the PEOU, PU, and sociodemographic data administered by the RAs.

During each clinic follow-up, the participants repeated the e-EQ-5D-5L/VAS, and the PEOU and PU questionnaire. The patient’s longitudinal e-EQ-5D-5L/VAS report which showed the change in scores since his/her initial visit was given to the doctor, and both the doctor completed their PEOU and PU questionnaire after the consultation.

Data collection occurred between June 1, 2020 and December 31, 2021. In total, 49 doctors and 665 patients were recruited from three clinics.

Study Instruments.

##### Chinese (Hong Kong) e-EQ-5D-5L/VAS

The EQ-5D-5L comprises of five questions assessing five HRQOL dimensions (mobility, self-care, usual activities, pain/discomfort, and anxiety/depression). Each question has five response options (from no problems to extreme problems) and is converted to a composite utility score from zero (death) to one (perfect health), with a scoring algorithm derived from a population-based valuation. A validated Chinese (Hong Kong) version of the EQ-5D-5L is available and the Hong Kong population-specific EQ-5D-5L value set has been developed [42]. VAS measures global health from zero (worse health) to 100 (best health). An online platform was developed by the team of author CO to administer the Chinese e-EQ-5D- 5L/VAS, to calculate the utility score, and to generate a report on the longitudinal EQ-5D-5L profile scores, utility scores, and VAS scores.

##### Patient PEOU and PU questionnaire

Patients’ perceptions of the e-EQ-5D-5L/VAS was assessed by using the 4-item PEOU and 4-item PU questionnaire, adapted from the Chinese PEOU and PU questionnaire validated by Yan et al. [36] (Additional file 1: Appendix 3). Each item was rated on a five-point Likert Scale (1. strongly disagree to 5. strongly agree). Summative PEOU and PU scores were calculated by adding the item scores, which had an Cronbach’s alpha of 0.90 and 0.89, respectively, supporting internal reliability [36].

##### Patient socio-demographic questionnaire

Data on socio-demographic characteristics of patients (age, sex, education level, marital status, and occupation) and clinical characteristics (number of chronic diseases, type of musculoskeletal disease, and duration of diagnosis) were collected.

##### Doctors’ PEOU and PU questionnaire

Doctors’ perceptions of the e-EQ-5D-5L/VAS report was assessed by using a 3-item PEOU and 2-item PU questionnaire (Additional file 1: Appendix 4). Each item was rated on a five-point Likert Scale (1. strongly disagree to 5. strongly agree) and summative scores were calculated.

##### Feasibility and acceptability outcome measures

The completion and response rates, and completion time, at baseline and follow-ups of the e-EQ-5D-5L/VAS were measured as indicators of feasibility among patients. The time for completion was recorded by the electronic platform. Feasibility for the doctors was measured by the doctor-reported “extra time” they spent on the consultation to review and address the patients’ reports. The PEOU and PU ratings by patients and doctors were used to indicate the acceptability of the technology.

### Individual and focus group interviews

From October to December 2021, semi-structured individual interviews for patients and separate focus groups for five doctors and three RAs were adopted to triangulate data. Purposeful sampling of RAs, doctors and patients was done across the three clinics. Two doctors who had rated on the largest number of EQ-5D/VAS reports in each clinic, with a total of six, were invited to join a focus group interview, and five accepted. Among the seven RAs who had assisted the administration of the e-EQ-5D in the clinics, three who were still working in the study clinics (one in each clinic) at the time of the qualitative study participated in the focus group interview. Patients’ age, education and number of chronic diseases were varied. The patient interviews were carried out by two trained RAs. Authors (AN & KL) experienced in qualitative research moderated the focus group interviews.

Topic guides (Additional file 1: Appendix 5) were developed for the semi-structured individual interviews and focus groups. Patient interviews lasted for 10–15 min and were conducted privately in Cantonese. The interviewer wrote the responses verbatim. The RA and doctor focus group interviews were video-recorded over Zoom, lasted for approximately 30 min each, and were conducted in Cantonese and English, respectively.

### Data analysis

#### Quantitative

Descriptive statistics were used to present the patient baseline characteristics, e-EQ-5D-5L/VAS completion rate and time, PEOU and PU ratings and summative scores, and doctor’s time. Response rate was calculated by dividing the number of patients who had repeated the e-EQ-5D-5L/VAS by the total number of patients who had attended the follow-up in the clinics.

Differences in the completion time of e-EQ-5D-5L by the patient and doctor’s time spent on interpreting e-EQ-5D-5L, and summative scores of PEOU and PU between baseline and the two follow-ups were tested by repeated measure ANOVA. The differences in PEOU (agree/strongly agree to all items) and PU (agree/strongly agree to at least one item) ratings across three time-points were tested by chi-square test. Multiple linear regression was carried out to evaluate whether the patients’ socio-demographics, clinical characteristics, baseline e-EQ-5D-5L utility score and VAS score predicted the PEOU and PU outcomes of patients and doctors. All statistics and figures were generated with the use of IBM SPSS Statistics 17. P-value of less than 0.05 was considered as statistically significant.

#### Qualitative

The transcripts of patient interviews and RA focus groups were transcribed and translated into English by two authors (JC and WC). The doctor focus group was transcribed verbatim by two RAs. The accuracy of the transcription and translation was checked independently by one of the authors (AN or KL). A thematic approach for the qualitative data analysis was used. The interview transcripts were independently coded by two authors (AN and KL). The reliability and validity of the analysis and interpretation were assessed by checking the coding consistency between the two sets. Inconsistencies were resolved by discussion between the two authors to reach an agreement on a common theme.

## Results

### Quantitative results

**Fig. 1:**
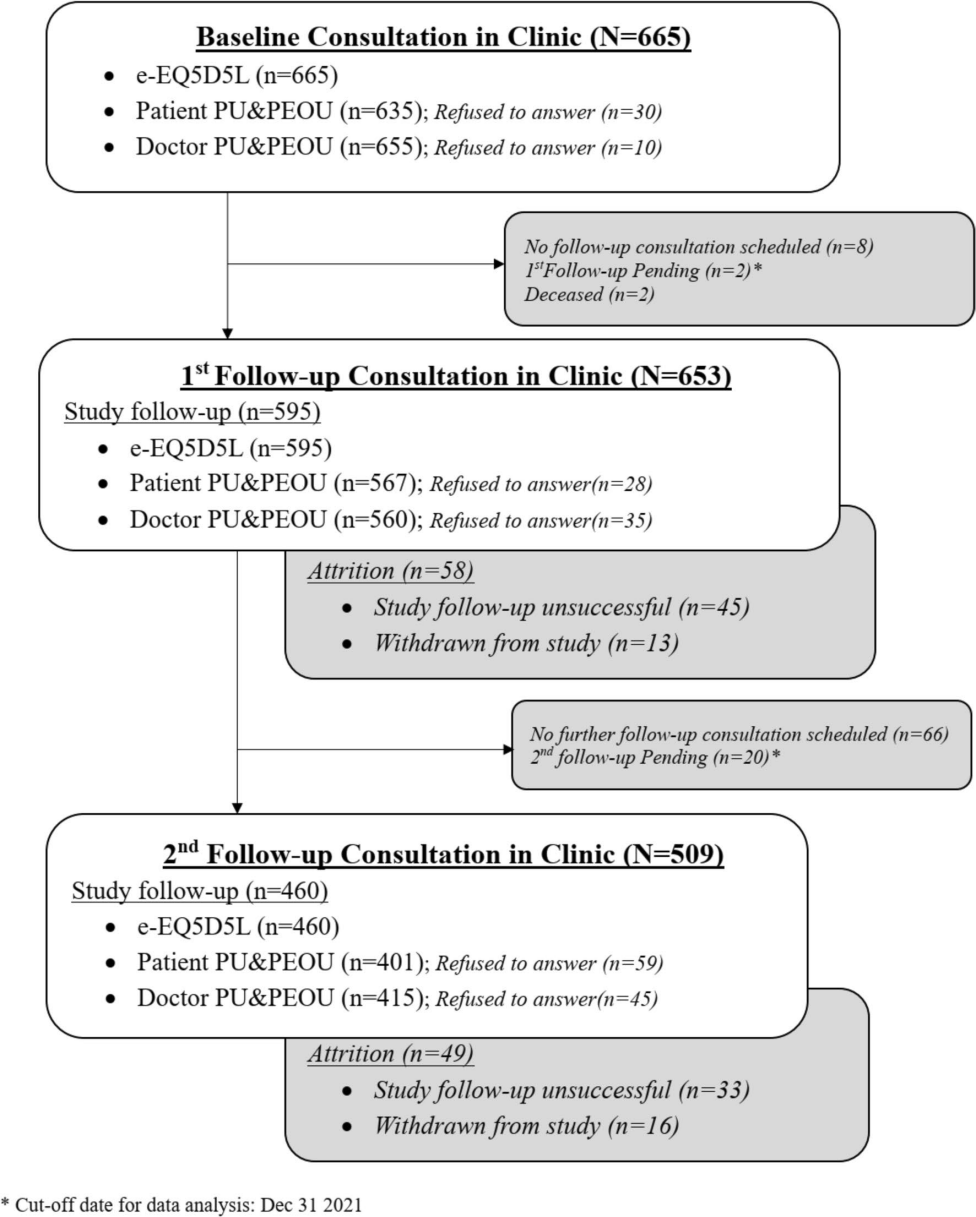
Study participant flow chart

The study participant flow chart is shown in Fig. 1. Baseline characteristics of the patients are shown in Table 1. Mean patients’ age was 68.74 (SD = 10.18). 58.4% of the participants had primary school education or below and 65.7% had knee problems only. The mean baseline EQ-5D-5L utility and VAS scores were 0.66 (SD = 0.28) and 64.01 (SD = 18.20), respectively.

**Table 1:** Baseline characteristics of subjects (N = 665)

Characteristics	n	%
*Socio-demographic*		
Gender	(665)	
Male	204	30.7
Female	461	69.3
Age (years old)	(665)	
18–50	20	3.0
51–60	99	14.9
61–70	270	40.6
71–80	183	27.5
81 or above	93	14.0
Education	(664)	
None received/Primary	388	58.4
Secondary	232	35.0
Tertiary or above	44	6.6
Marital status	(663)	
Never married/Separated/Divorced/Widowed	173	26.1
Married	490	73.9
Occupation	(618)	
Unemployed/Retired/Homemaker	522	84.5
Labour work	55	8.9
clerical work	22	3.6
Professional or manager	19	3.0
*Clinical*		
Types of musculoskeletal disease	(665)	
Back only	154	23.2
Knee only	437	65.7
Both	74	11.1
Duration of musculoskeletal disease	(658)	
< 1 year	101	15.3
1–5 years	187	28.4
5–10 years	137	20.8
> 10 years	233	35.5
Number of the comorbidity	(665)	
0	81	12.1
1	305	45.9
2	187	28.1
3	71	10.7
4 +	21	3.2
Comorbidities	(299)	
Hypertension only	272	91.0
Diabetes mellitus only	27	9.0
Both hypertension and diabetes	158	52.8
*EQ-5D-5L response distribution*		
Mobility	(665)	
No problem	276	41.5
Slight problems	215	32.3
Moderate problems	114	17.1
Severe problems/Unable to	60	9.0
Self-care	(665)	
No problem	524	78.8
Slight problems	94	14.1
Moderate problems	29	4.4
Severe problems/Unable to	18	2.7
Usual activities	(664)	
No problem	359	54.1
Slight problems	175	26.4
Moderate problems	91	13.7
Severe problems/Unable to	39	5.9
Pain/Discomfort	(665)	
No problem	83	12.5
Slight problems	297	44.7
Moderate problems	197	29.6
Severe problems/Unable to	88	13.2
Depression/anxiety	(665)	
No problem	359	54.0
Slight problems	203	30.5
Moderate problems	63	9.5
Severe problems/Unable to	40	6.0
EQ-5D-5L/VAS scores (mean, SD)	(664)	
Utility score (range: − 0.8637 to 1)	0.66	0.28
VAS score (out of 100)	64.01	18.20

EQ-5D-5L = EuroQoL 5-Dimension 5-Level; *VAS* Visual Analogue Scale

Feasibility and acceptability results are shown in Table 2. The e-EQ-5D-5L/VAS completion rates, the primary outcome of feasibility, were 99.8% and 100.0% at baseline and follow-ups, respectively. The response rates at follow-ups were 90.0%-91.1%. The mean completion time by patients significantly decreased from baseline (120.66 ± 110.74 s) to first (83.99 ± 57.16 s) and second follow-ups (105.22 ± 82.93 s) (*p* < 0.001). There was a significant drop in the time the doctors needed to interpret and address the report by 0.36 min from baseline (2.02 ± 1.51 min) to follow-ups (*p* = 0.006).

**Table 2:** Feasibility and acceptability of e-EQ-5D-5L/VAS at baseline and follow-up

Patient perspective	Baseline	1st follow-up	2nd follow-up	*p*-value
Feasibility	N = 665	N = 595	N = 460	–
Completion rate^a^ of e-EQ-5D-5L/VAS (% (n))	99.8 (664)	100.0 (595)	100.0 (459)	–
Response rate^b^ of e-EQ-5D-5L	100%	91.11%	90.00%	
Time to complete e-EQ-5D-5L/VAS (in seconds) (mean ± SD)^§^	120.66 ± 110.74 (665)	83.99 ± 57.16 (544)#	105.22 ± 82.93 (334)#	< 0.001
Perceived Ease of Use	N = 635	N = 567	N = 401	
Overall Agree/Strongly agree to all items (% (n))^†^	37.2 (236)	46.9 (266)	47.1(189)	< 0.001
Summative score (mean ± SD, out of 20)^§^	12.26 ± 3.82	13.00 ± 3.57	13.22 ± 3.27	< 0.001
Perceived Usefulness	N = 635	N = 567	N = 401	
Agree/Strongly agree to at least one item (% (n))^†^	85.5 (543)	86.9 (493)	85.3 (342)	0.221
Summative score (mean ± SD, out of 20)^§^	14.65 ± 2.37	14.78 ± 2.45	14.49 ± 2.68	0.173

Doctor perspective	Baseline (N = 655)	1st follow-up (N = 560)	2nd follow-up (N = 415)	*p*-value
Extra time spent on interpreting e-EQ-5D-5L/VAS (in minutes) (mean ± SD)^§^	2.02 ± 1.51	1.72 ± 1.28	1.66 ± 2.00	0.006
*Perceived ease of use*				
Overall Agree/Strongly agree to all items (% (n))^†^	76.2 (499)	79.3 (444)	85.1 (353)	< 0.001
Summative score (mean ± SD, out of 10)^§^	7.74 ± 1.07	7.83 ± 1.20	7.95 ± 1.10	0.007
*Perceived usefulness*				
Agree/Strongly agree to at least one item (% (n)) ^†^	72.0 (471)	69.2 (387)	72.0 (299)	0.160
Summative score (mean ± SD, out of 15)^§^	10.89 ± 1.93	11.14 ± 2.14	11.22 ± 2.07	0.022

e-EQ-5D-5L/VAS electronic five-level: Euroqol 5 dimension and visual analog scale

^a^Completion rate = the number of e-EQ-5D-5L/VAS that were fully answered divided by the total number of attempted e-EQ-5D-5L/VAS

^b^Response rate = the number of subjects who had repeated the e EQ-5D-5L/VASs divided by the total number of subjects who had attended the follow-up in the clinics

^†^Chi-square was used for analysis

^§^Repeated measure ANOVA was used for analysis, which only included subjects with both valid Baselines, 1st follow-up and 2nd follow-up data;

^#^The data on completion time of e-EQ-5D-5L/VAS during follow-ups were available only for subjects followed up after 23 March, 2021

The proportion of patient participants that agreed to all PEOU questions statistically improved over time from 37.2% to 47.1% (*p* < 0.001). The summative PEOU score improved by 0.96 over time with a bigger change between baseline (12.26 ± 3.82) and first follow-up (13.00 ± 3.57) (*p* < 0.001). The proportion of patients who agreed to at least one PU question was similar at baseline and follow-ups (85.3–86.9%). There was no statistical difference in PU summative scores over time. The doctors agreed to both PEOU questions for a great majority (76.2–85.1%) of the patients’ reports. The summative score for doctors’ PEOU significantly improved over time (*p* = 0.007). Doctors’ agreeing to at least one PU item was stable (69.2–72.0%). Conversely, the summative PU score significantly increased from baseline (10.89 ± 1.93) to first (11.14 ± 2.14) and second follow-ups (11.22 ± 2.07) (*p* = 0.022).

Table 3 shows the factors associated with patients' PEOU and PU scores at baseline. Multiple linear regression for patients showed that older age (β = − 0.230; *p* < 0.001) and having primary or below (β = − 0.453; *p* < 0.001) and secondary school education (β = − 0.158; *p* = 0.032) compared to tertiary education or above was related to lower baseline summative PEOU score. Being a labour worker was statistically related to better PEOU (β = 0.123; *p* = 0.008), but being unemployed/homemaker/retired was related to better PU scores (β = 0.137; *p* = 0.027) compared to being a professional or manager. Lower utility score was significantly correlated with higher PEOU scores (β = − 0.111; *p* = 0.005), whereas higher VAS score was associated with better PEOU (β = 0.089; *p* = 0.025) and PU scores (β = 0.090; *p* = 0.044). Table 4 shows the factors associated with doctors’ PEOU and PU scores. Patient being a clerical worker was related to better doctors’ PEOU (β = 0.104; *p* = 0.021) and PU (β = 0.132; *p* = 0.003) compared to being a professional or manager.

**Table 3:** Factors associated with patients’ PEOU and PU scores on the e-EQ-5D-5L/VAS at baseline

Variable	Patient PEOU		Patient PU	
	Baseline summative score		Baseline summative score	
	Standardized β-coefficient	*p*-value^†^	Standardized β-coefficient	*p*-value^†^
*Socio-demographic*				
Gender				
Male	0.021	0.580	− 0.064	0.138
Female^§^	–	–	–	–
Age	− 0.230	< 0.001*	− 0.061	0.208
Education				
None received/Primary	− 0.453	< 0.001*	− 0.129	0.139
Secondary	− 0.158	0.032*	− 0.041	0.628
Tertiary or above^§^	–	–	–	–
Occupation				
Unemployed/Homemaker/Retired	0.049	0.362	0.137	0.027*
Labour work	0.123	0.008*	0.066	0.208
Clerical work	0.076	0.058	0.066	0.454
Professional or manager^§^	–	–	–	–
Marital Status				
Never married/Separated/Divorced/Widowed^§^	–	–	–	–
Married	0.038	0.303	− 0.034	0.454
*Clinical characteristic*				
Number of chronic diseases	0.038	0.282	− 0.046	0.272
Type of musculoskeletal disease				
Back only	0.019	0.599	0.009	0.819
Knee only	0.054	0.136	< 0.001	0.994
Both^§^	–	–	–	–
Duration of diagnosis				
< 1 year^§^	–	–	–	–
1–5 years	− 0.006	0.906	− 0.048	0.245
5–10 years	0.058	0.231	− 0.054	0.352
> 10 years	0.002	0.967	− 0.035	0.527
e-EQ-5D-5L utility score	− 0.111	0.005*	− 0.019	0.741
VAS score	0.089	0.025*	0.090	0.044*

e-EQ-5D-5L/VAS electronic five-level Euroqol 5 dimension and visual analog scale

*VAS* visual analogue scale, *PEOU* perceived ease of use, *PU* perceived usefulness

^§^Reference category

^†^Multiple linear regression was used for analysis

*Statically significant with *p* < 0.05

**Table 4:** Factors associated with doctors’ PEOU and PU scores on the e-EQ-5D-5L/VAS report at baseline

Variable	Doctor PEOU		Doctor PU	
	Baseline summative score		Baseline summative score	
	Standardized β-coefficient	*p*-value^†^	Standardized β-coefficient	*p*-value^†^
*Socio-demographic*				
Gender				
Male	− 0.051	0.233	− 0.069	0.103
Female^§^	–	–	–	–
Age	− 0.009	0.843	0.059	0.213
Education				
None received/ Primary	0.023	0.787	0.047	0.588
Secondary	0.107	0.199	0.117	0.157
Tertiary or above^§^	–	–	–	–
Occupation				
Unemployed/Homemaker/Retired	0.004	0.950	− 0.009	0.876
Labour work	0.019	0.709	0.019	0.714
Clerical work	0.104	0.021*	0.132	0.003*
Professional or manager^§^	–	–	–	–
Marital status				
Never married/Separated/Divorced/Widowed^§^	–	–	–	–
Married	− 0.037	0.371	− 0.015	0.712
*Clinical characteristic*				
Number of the chronic diseases	0.059	0.146	0.075	0.064
Type of musculoskeletal disease				
Back only	− 0.030	0.466	− 0.037	0.371
Knee only	− 0.011	0.793	− 0.040	0.330
Both^§^	–	–	–	–
Duration of diagnosis				
< 1 year^§^	–	–	–	–
1–5 years	0.019	0.748	0.024	0.675
5–10 years	0.044	0.425	0.047	0.386
> 10 years	− 0.002	0.972	− 0.034	0.551
e-EQ-5D-5L utility score	0.039	0.377	− 0.009	0.843
VAS score	0.027	0.546	0.053	0.235

e-EQ-5D-5L/VAS electronic five-level Euroqol 5 dimension and visual analog scale

*VAS* visual analogue scale, *PEOU* perceived ease of use, *PU* perceived usefulness

^§^Reference category

^†^Multiple linear regression was used for analysis

*Statically significant with p < 0.05

### Qualitative results

The themes and subthemes of all the qualitative interviews and focus groups are shown in Tables 5 and 6. The characteristics of the participants in the qualitative study are shown in the Additional file 1: Table S1, and the key quotations by themes are shown in the Additional file 1: Tables S2 and S3 and Appendix 6.

**Table 5:** Themes and subthemes on feasibility and acceptability of the e-EQ-5D-5L/VAS synthesized from patient interviews and RA focus group

Themes	Subthemes	Source of information	
		Patients	Research assistants
*Ease of use in terms of methods of administration*			
Difficulty in using an e-platform	Technology-related problems	V	V
Difficulty of self-administration	Vision-related problems	V	V
	Requiring assistance to understand the survey question	V	V
	Literacy problems	V	
	Increasing age		V
Ease of use in terms of questions			
Difficulties in understanding	Unclear definition of the terms	V	V
	Improved understanding after repeated use	V	V
Difficulties in answering	Unable to describe own health in levels	V	V
	Unable to specify score due to fluctuating health conditions	V	V
	Providing a score range instead of an exact score		V
	Perceiving the response options in e-EQ-5D-5L/VAS as too “severe”		V
*Perceived usefulness*			
Usefulness to patients	Understand the patient situation	V	
	Helpful for treatment	V	
	Uncertain usefulness	V	
	Not useful to patient	V	V
Usefulness to others	Useful to researcher	V	
	Useful to other patients	V	
*Feasibility*			
Time for completing the e-EQ-5D-5L/VAS	Short completion time	V	V
	Feel like chatting	V	
	Difficulties with finding the patients in the clinic		V
	Slight impact by unstable network		V
	Limited time before consultation		V
Time for consultation	Time-saving by knowing their painful condition before the consultation	V	
Poor patient attitudes	Annoyance by repeated surveys		V
	Perceiving (the EQ-5D-5L information) useless		V

**Table 6:** Themes and subthemes on feasibility and acceptability of the e-EQ-5D-5L/VAS synthesized from doctor focus groups

Themes	Subthemes
*Ease of use in terms of viewing the report*	
Clarity of information	Clear layout of the report
Ease of interpretation	Easy to compare with the population mean
	Easy to see the trend of scores
Limitations in interpretation	Other confounders present
Positive feelings towards perceived usefulness	
Understand the patient better	Better understanding of impact on patient's daily living
	Monitoring the progress of MSK condition
	Useful for less active patients or patients with MSK issues as the chief complaint
	Prompting a discrepancy in pain perception between doctor and patient
Manage the patient better	Increased lifestyle management/counselling patients
	Selecting treatment based on the trend
*Negative feelings towards perceived usefulness*	
Situations where the report is not useful	Patients who already actively share about their MSK problems during consultation
	(Patient) Having follow-up by a specialist for MSK problem/ not coming for MSK issues
Aspects of care not addressed	Patient needs are better communicated verbally than by a score
*Feasibility*	
Time for interpretation	Quick reference
	Time-saving by knowing the patient’s pain condition before the consultation
Time to address the result	Balancing between the usefulness and additional time for addressing the MSK problem
	Limited consultation time/ The need in addressing other medical problems (in the same consultation)

#### Patients’ perspective

While some patients found the e-platform easy-to-use, some had problems using technology and self-administering the survey due to vision and literacy problems. The RAs attributed this due to advanced age. Therefore, some patients needed the RA’s assistance to comprehend the questions. While most patients easily answered the e-EQ-5D-5L/VAS questions, others felt that some terms and questions were unclear. Moreover, they expressed difficulty assessing their health on a level or specifying scores due to their fluctuating health conditions. Some had difficulty providing an exact score for the VAS, though they could often provide a range. The RAs also believed that few patients perceived some of the response options as “too severe” to be selected. Encouragingly, RAs and patients agreed that understanding of the questions improved after repeated use. Also, most patients felt that the e-EQ-5D-5L/VAS helped the doctors to understand their clinical situation, particularly their pain. A few patients thought doing the e-EQ-5D-5L/VAS helped the researchers and other patients indirectly and their own treatment. However, some were unsure about the PU and suggested that it was more useful to the doctors than for themselves. Other patients found it not useful because the e-EQ-5D-5L/VAS did not lead to treatment changes and sometimes the doctors did not look at the report. Reassuringly, patients felt the survey administration was quick and felt like chatting, and it saved time for doctors to know about their painful condition before the consultation. The RAs thought the completion time was short but sometimes they had difficulties with identification of the patients during the follow-up visits, network connection problem that would sometimes prolong the survey time, and limited time before the consultations because patients had to complete other tasks before seeing the doctor. Moreover, the RAs agreed that patient attitude affected the feasibility. Patients’ attitudes were initially positive, but with repeated administrations, some patients got annoyed or were reluctant to do it because they saw that their doctor did not review their report and therefore felt the survey was useless.

#### Doctors’ perspective

All doctors agreed that the report was clear and easy to interpret. However, some doctors did not know if the scores were specific to their musculoskeletal problem or were also reflective of other medical problems. The report helped doctors to understand and manage their patients better. Doctors used different parts of the report to help them to assess the impact on patients’ activities of daily living, to monitor the progression of the disease, and the report provides a gateway for discussion with patients who may not bring up the musculoskeletal problem or whose pain perception differed from the doctor’s. It helped to increase counseling for some patients and to select treatment. Doctors found the report less useful for patients who were forthcoming about their musculoskeletal problem, who did not have pain that day, or whose musculoskeletal problem was being followed by other healthcare providers. In addition, doctors commented that there were certain areas that the report was unable to address, such as patients' needs and expectations. Doctors thought that the report served as a quick reference and saved time because they knew about the pain condition early in the consultation. However, the biggest challenge was balancing the usefulness of the report and the additional time to address the result because most patients had other medical issues to be dealt with in the same consultation.

## Discussion

This study aimed to assess the feasibility and acceptability of using the electronic platform to collect and report HRQOL measured by the Chinese e-EQ-5D-5L.

### Patient’s perspective on feasibility

Quantitatively, feasibility was measured in three ways. Firstly, completion rates were greater than 99.8%. This is better than other EQ-5D studies done in elderlies where the completion rates were just above 90% [23] and exceeded our target of 90%. Secondly, the response rates on follow-up assessments were greater than 90%. Our results were comparable to those of the Swedish orthopedic registry, with a response rate of 86.1% at baseline and 90.2% at first follow-up [4]. The drop in our response rate was largely due to being unable to successfully find the patient to complete the e-EQ-5D-5L/VAS. Additionally, patients’ attitudes played a role as some were less willing to complete the surveys in subsequent visits because they did not think the survey could change their management or the doctor did not review their report. Thirdly, completing the e-EQ-5D-5L/VAS was quick with an average of less than two minutes, compared to less than five minutes for the paper e-EQ-5D-5L administered in elderlies [43]. The RAs commented that time would only be a barrier if the patient arrived late to their appointment as this would interfere with the patient’s time to perform pre-consultation activities.

### Patients’ perceptions

Over 80% of patients perceived the e-EQ-5D-5L/VAS useful, which supported acceptability of HRQOL measurement in routine primary care. However, more than half of the patients did not perceive the e-EQ-5D-5L/VAS easy-to-use. The barriers for our patients to the formation of positive perceptions of e-EQ-5D-5L/VAS included being unfamiliar with technology, and having problems with vision, literacy, and understanding survey questions and responses. In the RA views, these problems were related to the old age. The study population was mostly senior (mean age of 68.74 years old), and we found age was associated with worse baseline PEOU scores. A study in Hong Kong studying the perceptions and acceptance of gerontechnology also found that age was associated with PEOU, but the effects of PU were mediated by other factors [44]. In Hong Kong, only 65% of persons aged over 65 had a smartphone and 62% have used the internet in the past year [41] which may be a reason for patients being unfamiliar with technology. Also, half of the patient participants had primary school education or below, and lower education was a factor associated with worse baseline PEOU scores. Poor literacy would not only affect their ability to read, but also to understand the questions. A systematic review showed that elderlies have more problems with interpretation of questions and understanding the VAS [23]. Quantitatively and qualitatively, our data showed that patient’s PEOU improved from baseline to follow-up which highlight that with repeated use, patients can learn to use technology. Similarly, a Canadian study showed participants were able to learn how to complete an e-HRQOL questionnaire, despite only 35% having a more than high school education [45]. Our study showed that those who are labour workers found the e-EQ-5D-5L/VAS easier to use and those who are retired, unemployed or homemakers found it more useful than professionals and managers, suggesting the report could enhance the communication on their health conditions with the doctor for these groups. Lastly, this study showed that patients with worse utility scores found it easier to use; however, patients who had higher VAS scores thought that it was easier to use and more useful. The discrepancy could be differences in what the utility score and VAS measure: VAS represents the patient perspective, whereas the utility score represents the society’s perspective.

Unfortunately, there is a paucity of studies assessing the acceptability of the EQ-5D. A UK qualitative study assessing asthmatic patients’ views on the EQ-5D-5L found negative perceptions of the tool as many of the domains were irrelevant to asthmatic patients. This present study is quite different and focuses on musculoskeletal problems which is easier to interpret using the EQ-5D.

### Doctors’ perspective on feasibility of implementation

Although interpreting and addressing the e-EQ-5D-5L/VAS report was quick, usually taking less than two minutes, doctors felt that the time to fully address the results would be a barrier. The average length of a consultation in Hong Kong’s public clinic is approximately six minutes [46] and a majority of the patients have at least one other comorbidities that would need to be addressed at the same visit. While efforts are being made to increase consultation time in the public sector [47], the use of the e-EQ-5D-5L was felt to help to understand the patient’s pain in a shorter amount of time which may facilitate shorter length of the consultation. An additional two minutes to address musculoskeletal problems could save time from the need for a repeat consultation, which ultimately may reduce the doctors’ work burden.

### Doctors’ perceptions

Research shows that the acceptance of HRQOL assessment by clinicians depend on the familiarity of the tool and what it measures [7]. We discovered that doctors found the e-EQ-5D-5L/VAS reports easy-to-use and useful. Encouragingly, PU and PEOU scores improved with repetition, implying that with time doctors become more familiar with interpreting and using the report. The PU rate did not reach our 80% target but is better than those found in another study [48]. In a QOL study of oncology patients, only in 42% of the visits did the physicians find the data clinically useful; but this could be related to the different QOL tool used, clinical setting, and clinical diagnosis [48]. The doctors in our study used different aspects of the report to learn about the impact on the patient’s life, to help to monitor the disease progression, and to provide a gateway for discussion. Our data show that the doctors’ PU was better for those who were clerical workers compared to professionals and managers and for those with more chronic diseases. We can hypothesize that clerical workers’ administrative roles may make it easier for doctors to communicate the report results for this group. Doctors agreed that the report saves time which may explain why they find it more useful for those with multiple chronic illnesses as they can better prioritize the problems. Studies on HRQOL measurements in real clinical practice also confirm that the additional information helped to enhance communication and to facilitate the management of patients [45].

## Limitations

Firstly, the doctors’ data on PEOU and PU could be biased because specific doctors' perceptions of the e-EQ-5D-5L/VAS report could have biased the results as 665 patients were not equally allocated to the 49 doctors. Secondly, sampling of patients for the qualitative interviews may be skewed as those who would be more “annoyed” would likely not agree to be interviewed. Thirdly, in real clinical practice, existing staff would need to be trained to take the task of introducing the assessment to patients, which was a part of feasibility not assessed in this study. Fourthly, the findings were limited to the Chinese patients with knee or back problems, which may not be generalizable to non-Chinese populations and patients with other musculoskeletal problems.

### Future direction to enhance implementation

Patients should come to a designated clinic area earlier than their scheduled follow-up time to complete the survey and to have a staff teach them how to use the device and explain the questions the first time. Adding an audio function to read out questions and record answers, and increasing the font size of the questions [49] may help patients with visual and literacy problems. Software upgrades that allow patients to mark on a line for the VAS question rather than indicating a numerical score may be easier for patients. Simplifying each page by having a meaningful title and using easy-to-understand icons have shown to be helpful [49]. To improve relevance, only patients whose musculoskeletal problems are under the clinic’s care and not other health care providers should complete the e-EQ-5D-5L/VAS.

## Conclusion

We found the e-EQ-5D-5L/VAS a feasible and acceptable measurement of HRQOL of patients with chronic knee and back problems in routine primary care, providing real-time reporting and feedback on HRQOL to assist doctors’ management during the consultation. The acceptability by medical practitioners is crucial for the implementation of a new technology in real clinical practice. The doctors’ feedback on the EQ-5D-5L/VAS report is generally positive as a means to enhance the holistic understanding of patients’ condition and a quick reference to help prioritizing health problems that can potentially save time in the overall consultation. Over time, patients can learn to use the electronic surveys more easily and the doctors become more comfortable with interpreting HRQOL results, which can facilitate wider implementation. Patients and doctors use little time to complete and interpret the e-EQ-5D-5L/VAS, respectively. These study findings should encourage medical practitioners to apply e-EQ-5D-5L/VAS on the targeted patients for more patient-centred management. Nonetheless, the administration of the HRQOL survey can be made easier for patients with visual and literacy difficulties, and the lack of consultation time is an important barrier to be addressed.

## Supplementary Information

**Additional file 1:** Supplementary Materials on e-EQ-5D-5L platform, study questionnaires, interview guides and qualitative interview subject characteristics.

## Abbreviations

EQ-5D: EuroQol-5-dimension
e-EQ-5D-5L: Electronic five-level Euroqol 5 dimension
e-EQ-5D-5L/VAS: Electronic five-level Euroqol 5 dimension and visual analog scale
HRQOL: Health-related quality of life
RCT: Randomized controlled trials
PEOU: Perceived ease of use
PRO: Patient-reported outcome
PU: Perceived usefulness
TAM: Technology acceptance model
VAS: Visual analogue scale

## Declarations

### Availability of data and materials

The datasets used during the current study are available from the corresponding author on reasonable request.
