# Supplementary material for: Feasibility and acceptability of electronic EQ-5D-5L for routine measurement of HRQOL in patients with chronic musculoskeletal problems in Hong Kong primary care
Source: Health Qual Life Outcomes. 2022 Sep 20;20:137. doi: 10.1186/s12955-022-02047-0 (PMC9487025; doi:10.1186/s12955-022-02047-0)
Supplement: Supplementary file 1 — Additional file 1: Supplementary Materials on e-EQ-5D-5L platform, study questionnaires, interview guides and qualitative interview subject characteristics. [file 12955_2022_2047_MOESM1_ESM.docx]

**Supplementary material Appendix 1: Electronic EQ-5D-5L and EQ-VAS Completion Procedure with Screenshots**

| **Step 1 –Each subject is assigned a unique subject number with QR code to log into the system to complete the EQ-5D-5L and EQ-VAS online** | |
| --- | --- |
| **Screenshot 1** | **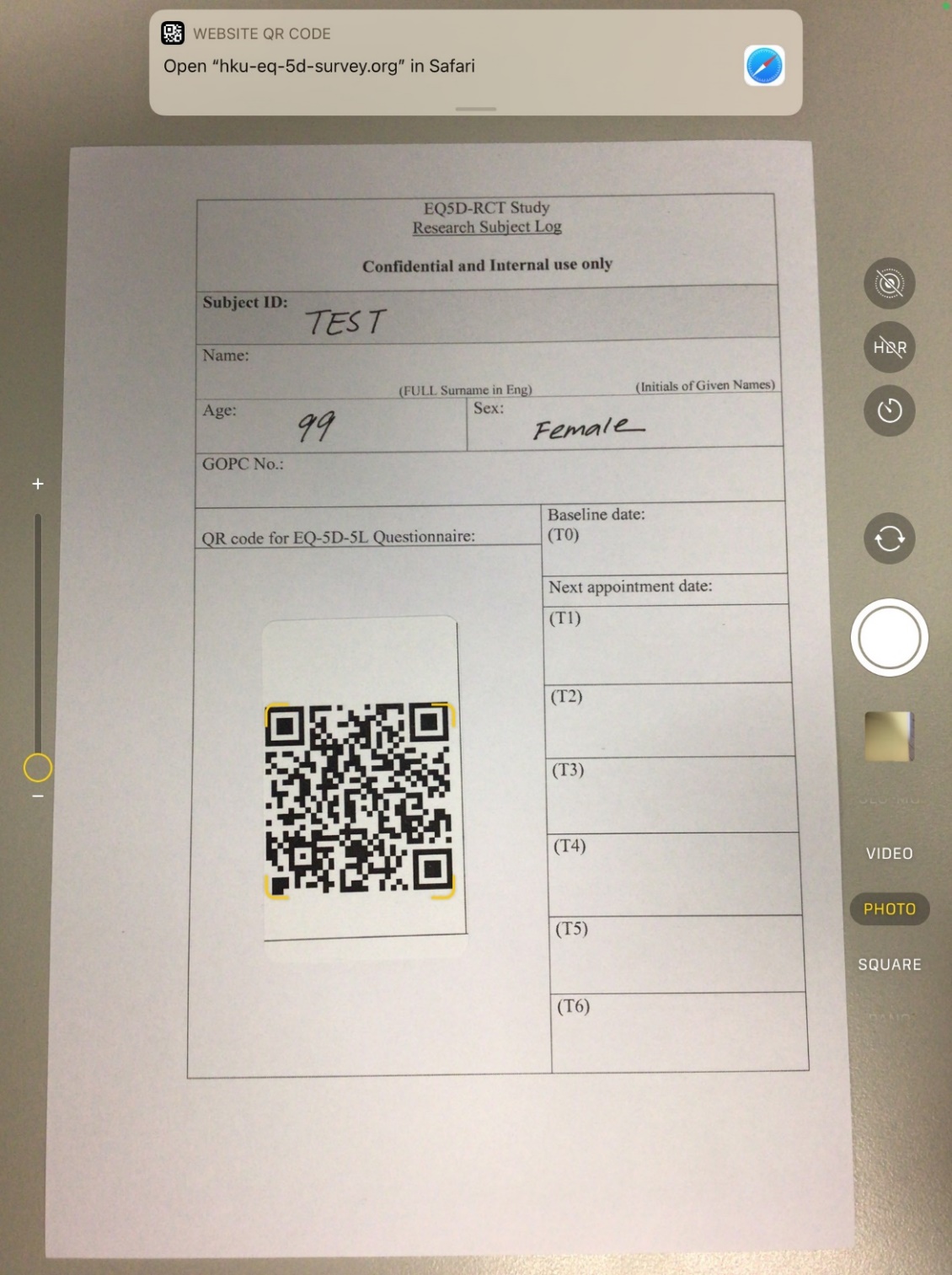** |

| **Step 2 – Enter the age and gender for linkage to the relevant Hong Kong age-gender specific population norm reference on the report** | |
| --- | --- |
| **Screenshot 2** | **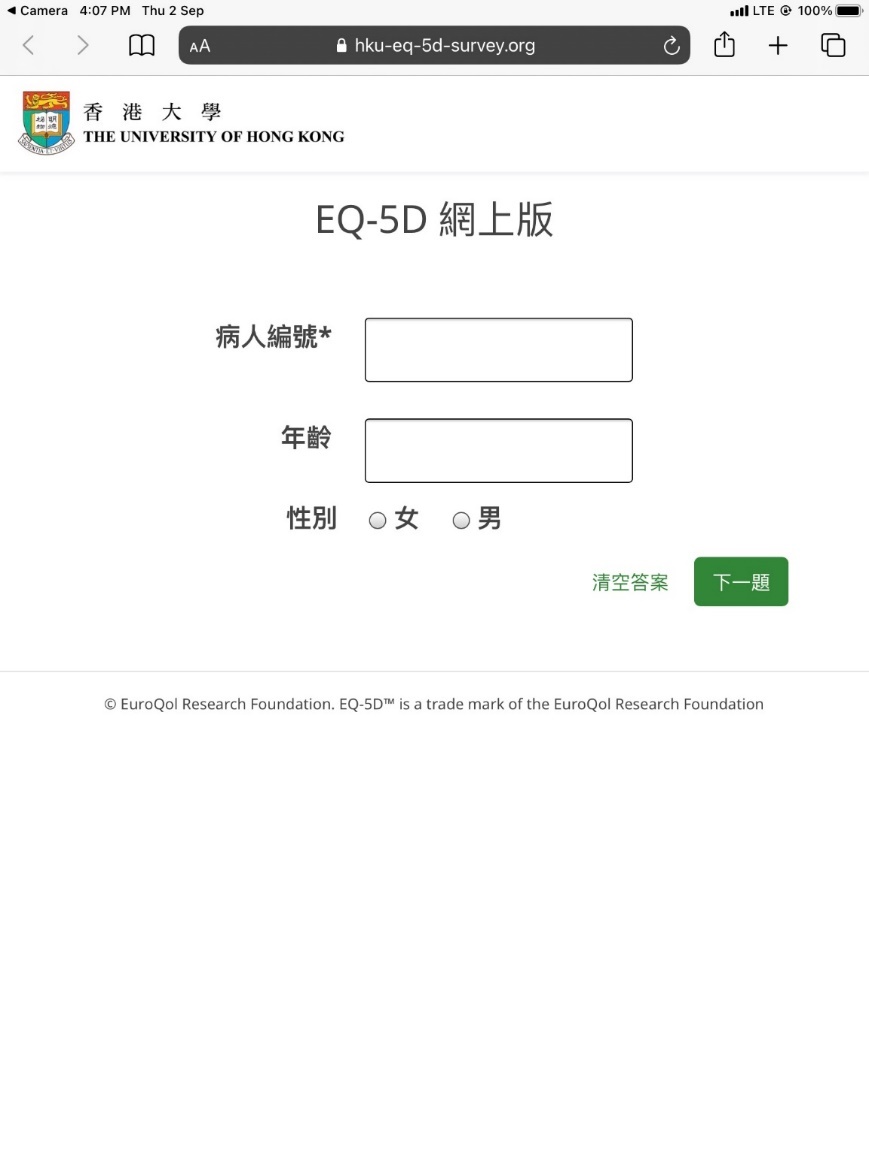**  “Age”  “Gender: Female; Male”  “Next Question”  “Subject number”  “Online EQ-5D” |

| **Step 3 –Completion of the EQ-5D item on Mobility**  **The instruction is shown before the item and the response options.** | |
| --- | --- |
| **Screenshot 3** | **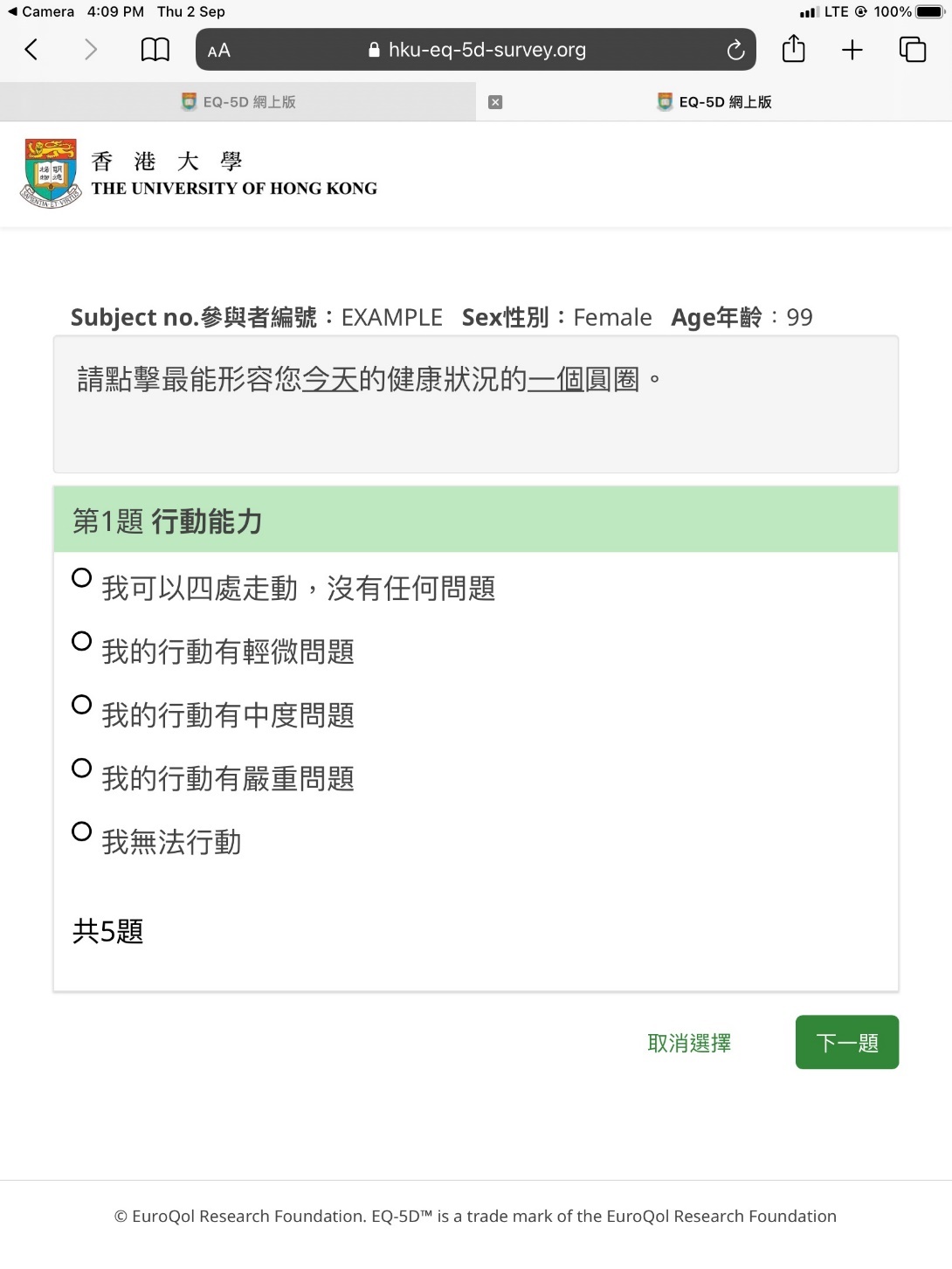**  “Please select the ONE statement that best describes your health today”  “Next Question”  “First Question: Mobility”  5 levels of response options |
| **N.B.** | **The subject needed to click “Next” before moving to the next item. The subject could choose not to provide an answer (i.e. skip the question) before moving to the next.** |

| **Step 4-7 – Completion of EQ-5D items on Self-care, Daily Activities, Pain and Emotion, each presented on one screen at a time with instruction shown before the item.** | |
| --- | --- |
| **Screenshot**  **4-7** | **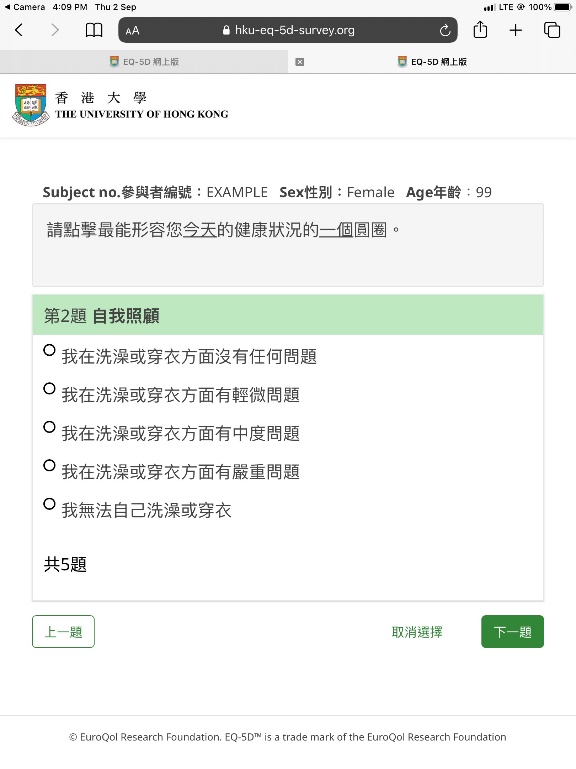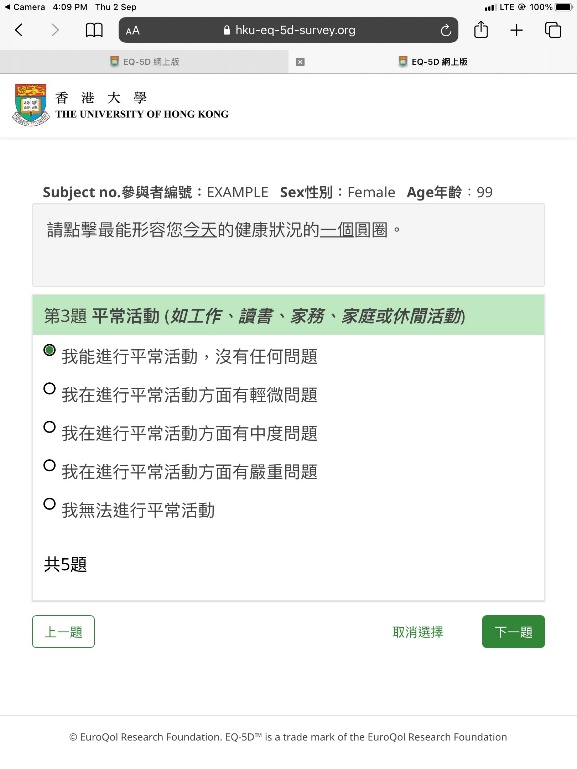**  “Third Question: Daily Activities”  “Second Question: Self-care”  **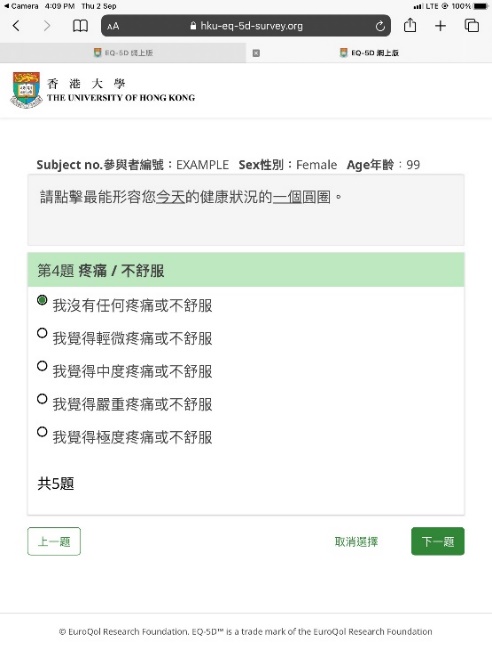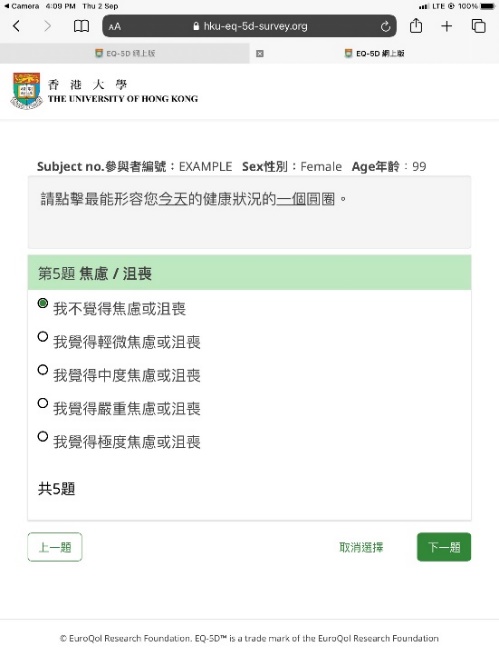**  “Fifth Question: Emotion”  “Fourth Question: Pain” |
| **N.B.** | **The subject needed to click “Next” before moving to the next item. The subject could choose not to provide an answer (i.e. skip the question) before moving to the next.** |

| **Step 8 – Completion of the EQ-VAS, the instruction is shown on the left and the subject needed to enter the appropriate number (0 to 100) in the box.** | |
| --- | --- |
| **Screenshot 8** | **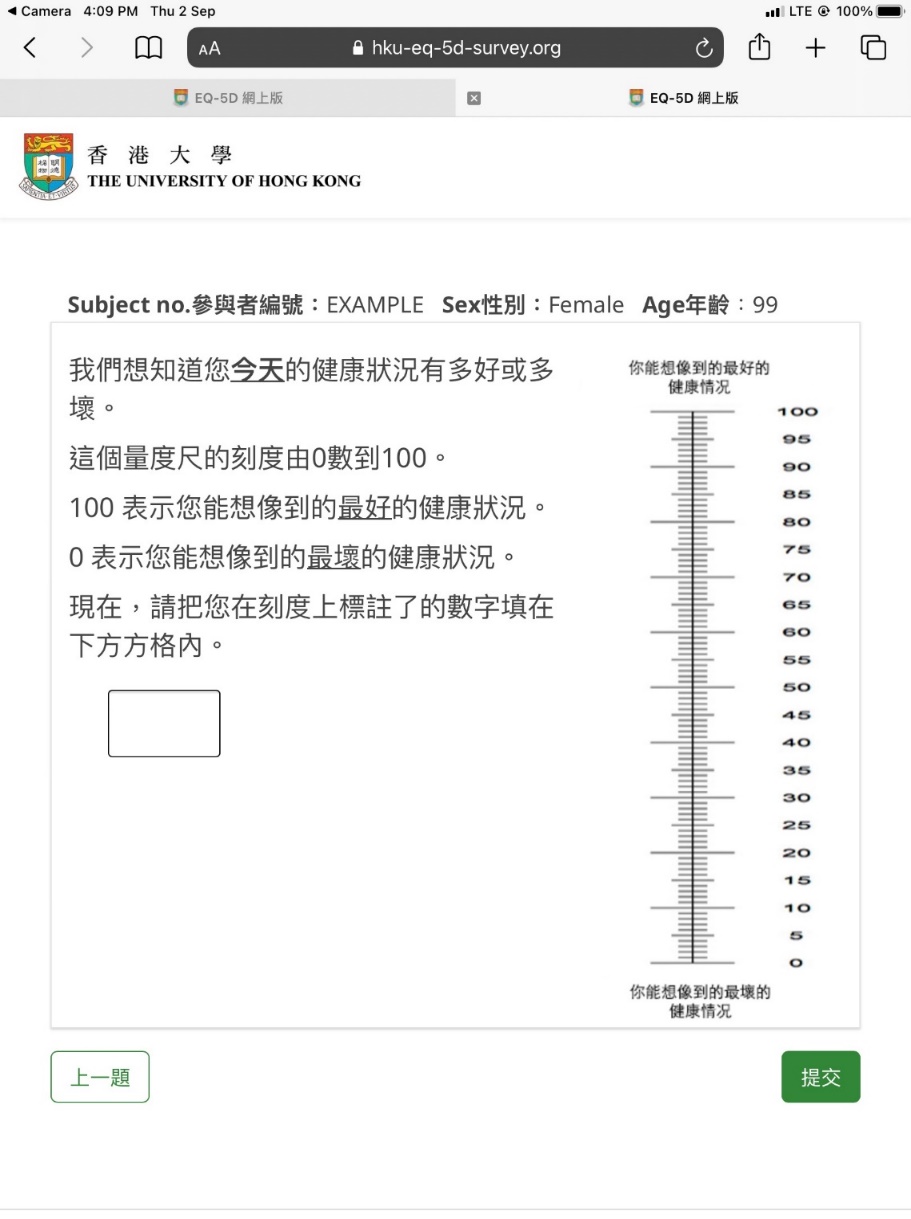**  “Submit”  Instructions on completing the EQ-VAS |
| **N.B.** | **the EQ-VAS was modified from the original 200mm to a 100mm scale from 0 (the worst imaginable health state) to 100 (the best imaginable health state), in order to fit into one page of the screen. To proceed, subjects needed to click “Submit” after this item.** |

| **Step 9 - Thank you – Your answers have been recorded** | |
| --- | --- |
| **Screenshot 9** | **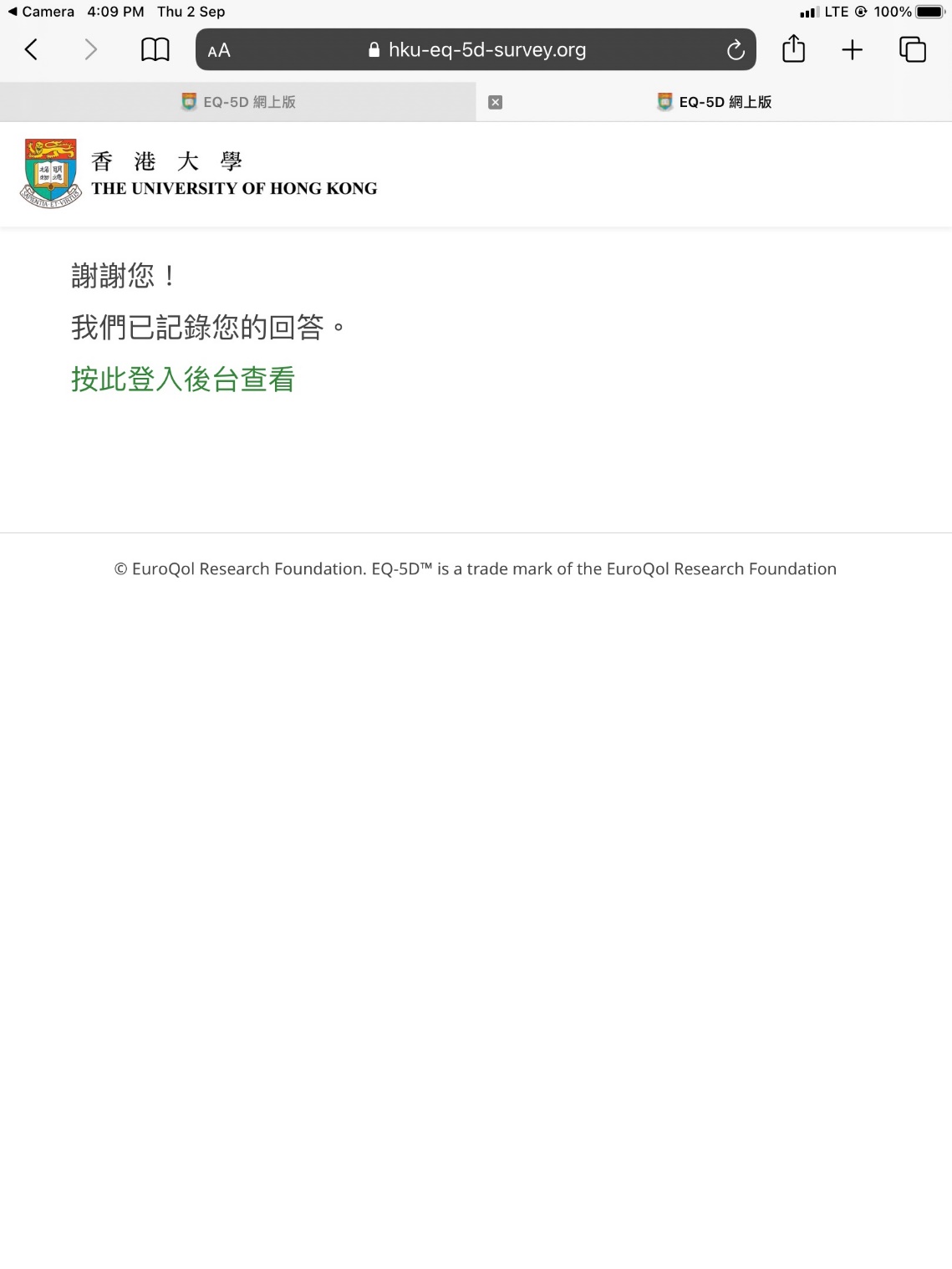**  “Thank you!”  “Your answers have been recorded”  “Click here to the login page of the server (for research staff only)” |
| **N.B.** | **After the subject has completed the electronic EQ-5D-5L and EQ-VAS, a report on the EQ-5D-5L, utility and VAS scores would be generated within one minute.** |

| **Step 10 – A Trained Research Assistant then Login the Sever to Retrieve the EQ-5D Report** | |
| --- | --- |
| **Screenshot 10** | **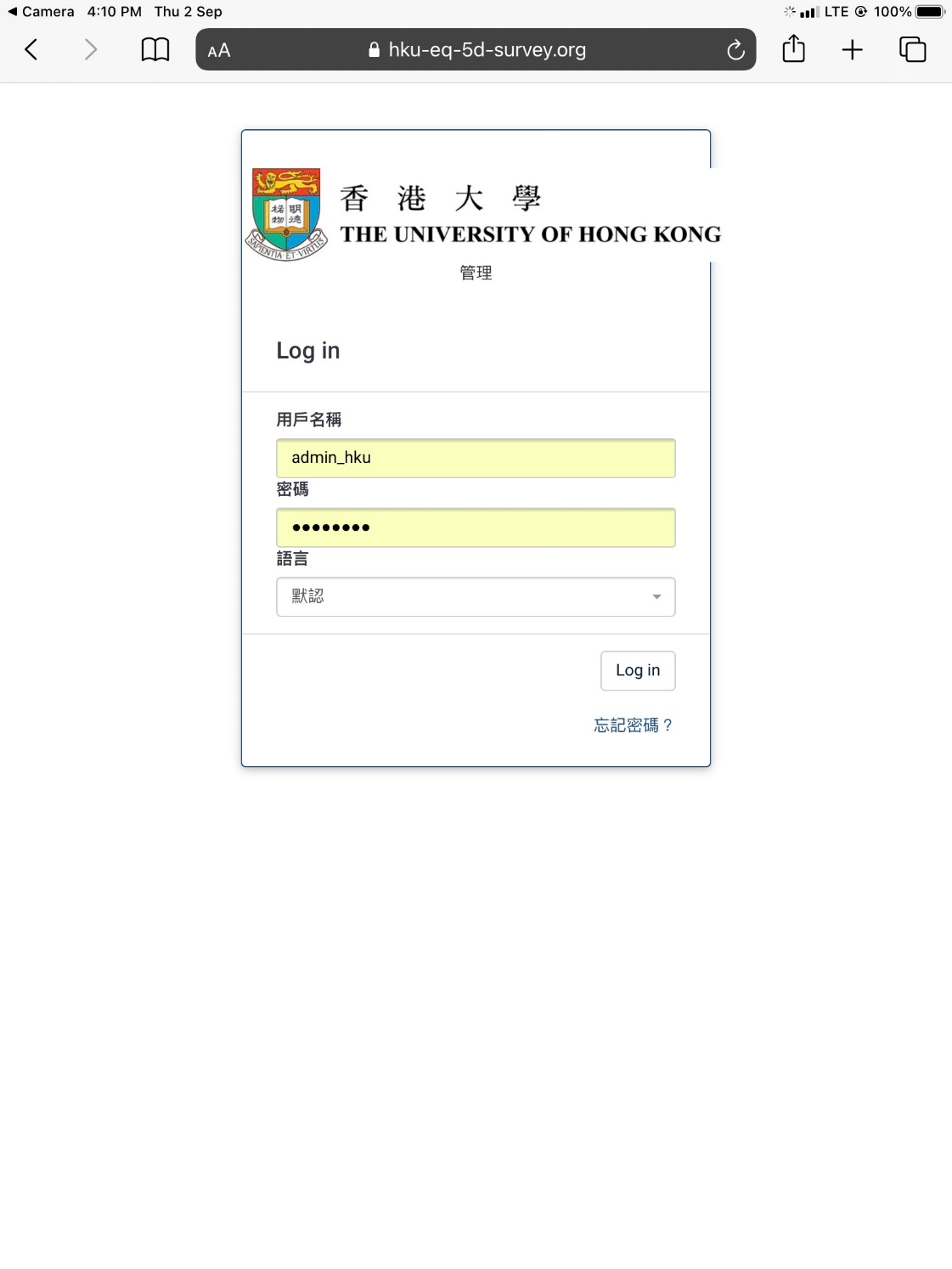** |
| **N.B.** | **To ensure data privacy and protection, credentials and password, which are only available to the study team, were required to log into the server** |

| **Step 11 –Printable EQ5D report** | |
| --- | --- |
| **Screenshot 11** | **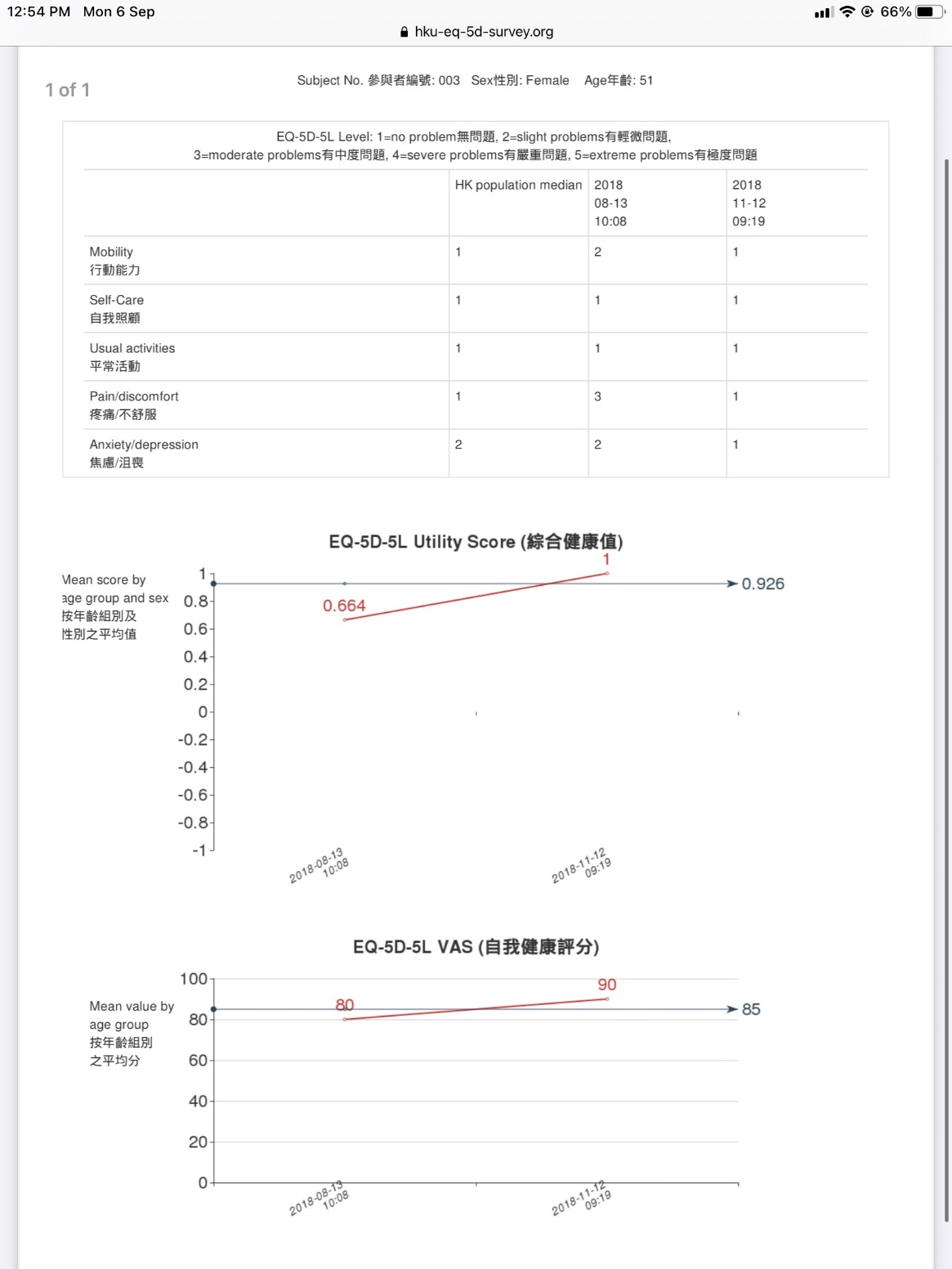** |
| **N.B.** | **The report included the subject’s longitudinal EQ-5D dimension, utility and VAS scores (up to five records), and the Hong Kong age-gender specific population norm reference.** |

**Supplementary Material Appendix 2: e-EQ-5D-5L Results Printout**
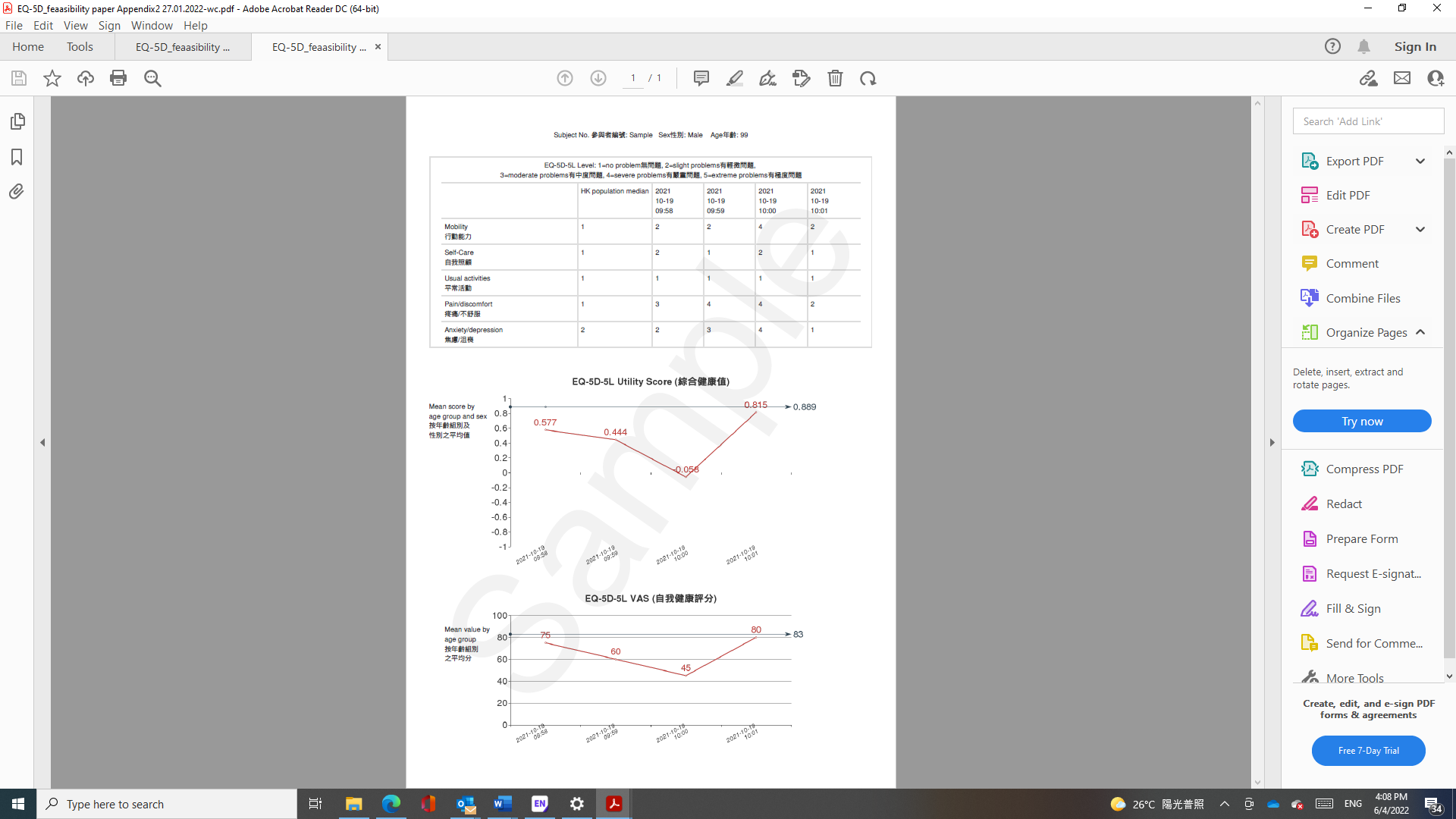


**Supplementary Material Appendix 3: Patient Perceived Ease of Use and Perceived Usefulness Questionnaire**

Please ✓ the appropriate answer for each question

| **Understanding of the questionnaire** | Strongly Disagree | Disagree | Neutral | Agree | Strongly Agree |
| --- | --- | --- | --- | --- | --- |
| 1. I can understand every question |  |  |  |  |  |
| 1. I can easily answer each   question |  |  |  |  |  |
| 1. Is there anything about your life apart from the EQ-5D that you want to tell your doctor?   _____________________________________________________________________________ | | | | | |
| **Perceived ease of use** | Strongly Disagree | Disagree | Neutral | Agree | Strongly Agree |
| 1. Learning to use the electronic platform is easy for you. |  |  |  |  |  |
| 1. You find it easy to get the   electronic platform to do what you want it to do. |  |  |  |  |  |
| 1. It is easy for you to become   skillful at using the electronic platform. |  |  |  |  |  |
| 1. You find the electronic platform easy to use. |  |  |  |  |  |
| **Perceived usefulness** | | | | | |
| 1. Using the electronic platform   improves your ability to manage your disease |  |  |  |  |  |
| 1. Using the electronic platform   helps you save time in managing your disease. |  |  |  |  |  |
| 1. Using the electronic platform enhances your effectiveness in managing your disease |  |  |  |  |  |
| 1. You find the electronic platform to be useful in managing your disease |  |  |  |  |  |

**Supplementary Material Appendix 4: Doctor’s Perceived Ease of Use and Perceived Usefulness Questionnaire**

**Perceived usefulness of the EQ-5D-5L report**

(Please ✓ the appropriate answer for each question)

| **Perceived usefulness of the EQ-5D report** | **Strongly Agree** | **Agree** | **Neutral** | **Disagree** | **Strongly Disagree** |
| --- | --- | --- | --- | --- | --- |
| 1. The EQ-5D data report is clear |  |  |  |  |  |
| 1. The EQ-5D data report is easy to understand |  |  |  |  |  |
| 1. The EQ-5D data report helps me to understand the patient’s needs better |  |  |  |  |  |
| 1. Using the EQ-5D-5L data report helps the management for my patient |  |  |  |  |  |
| 1. I wish to have the EQ-5D data report of the patient available to me in future consultations |  |  |  |  |  |
| 1. How much extra time did you spend in the consultation to review the patient’s EQ-5D-5L report and address the results?   ____________________ minutes | | | | | |
| 1. Apart from the domains included in the EQ-5D, is there any other aspect of the patient’s quality of life that you would like to collect before the patient sees you?   Please specify: ________________ | | | | | |

**Supplementary Material Appendix 5: Topic Guides for Qualitative Interviews and Focus Groups**

1. **Doctor Focus Group Interview Guide on Perceived Ease of Use and Usefulness of the Electronic EQ-5D-5L Report**

**Doctor participants name (clinic)**

1 ______________________________________________________

2 ______________________________________________________

3 ______________________________________________________

4 ______________________________________________________

5. _____________________________________________________

6. ______________________________________________________

Date: _____________________

1. **How did you find the EQ-5D-5L report?**

How did you use the report? What part of the report did you use?

**2. Did you gain additional information from the EQ-5D-5L report?**

If yes, what additional information did you gain? How did this additional information affect your management of the patient?

**3. Did you find the EQ-5D-5L report useful?**

If Yes: a. What is useful? b. How is it useful? c. In what situation was it useful and what situation do you think it was not useful? d. Did you use the longitudinal data in the follow up sessions; and if so how did you use it? (can show the F/U sample and ask about each time point)

If No: a. What is not useful? b. Why is it not useful? c. What would make it more useful?

How did you interpret the word “neutral” in the clinician usefulness questions?

1. For example, for 30% of the patients, doctor selected neutral for “The report helps me understand the patient’s needs better? For which group of patients was the EQ-5D-5L did it help to understand the patients’ needs better? For which group of patients did it not help?
2. For 42% of patients at baseline and 37.5% at follow up, doctor selected neutral for “The report helps the management for my patient? For which group of patients did the EQ-5D-5L help to manage the patient? For which group of patients did it not help to manage?

**4. Did you find the EQ-5D-5L report clear?**

If No: a. What is unclear? b. Why is it unclear?

If Yes: How did you interpret the report? What did you look at VAS vs utility score vs dimensions?

**5. Did you find the EQ-5D-5L report easy to understand?**

If No: a. What is hard to understand? b. Why is hard to understand?

**6. Do you want to have the EQ-5D-5L report of your patients with musculoskeletal problems regularly available to you at their consultations?**

If Yes: a. why ? b. How often do you want it?

If Not: a. why not?

How did you interpret the word “neutral” in the question “do you wish to have the report of the patient available to me in future consultations? 37% selected neutral for this question at baseline and at follow up?

Do you want to have the EQ-5D – 5L assessment on demand?

**7. Is there anything else you want to share about the EQ-5D-5L?**

Thank you for your support in the project and attending the focus group today. Feel free to contact us for any further feedback.

1. **RA Focus Group Interview Guide on Acceptance and Ease of Use of the Electronic EQ-5D-5L by Patients**

Date: ______________________

**Interview guide:**

* Please describe the logistics of the whole process from administering the electronic EQ-5D-5L questionnaire to preparing the report for the doctor.

1. What assistance did the patients need in completing the EQ-5D-5L questionnaire?

2. What proportion of patients required your assistance in completing the electronic EQ-5D-5L?

a. at baseline b. at first follow up c. at second & later follow up

3. Did you find the patients have any difficulty in completing the electronic EQ-5D-5L questionnaire?

If yes:

a. What was the difficulty?

b. Was there any question particularly difficult for the patient to complete? (If yes, which one and why?)

c. Did the patients have difficulty completing the VAS? (if yes, what was the difficulty?)

4. Did you encounter any difficulty with the electronic platform?

If yes: What was the difficulty?

5. Did you encounter any difficulty with the printing of the report?

If yes: What was the difficulty?

** What were the possible difficulties to ensure a smooth logistics of the whole process?

1. **Patients Feedback Interview Guide on Ease of Use and Usefulness of the Electronic EQ-5D-5L**

Subject number: _____________ Gender _________ Age ___________ Date: __________________

1. Did you find completing the electronic EQ-5D-5L questionnaire difficult? _____________

If yes:

a. What was the difficulty? __________________________________________________

__________________________________________________________________________

___________________________________________________________________________

b. Did you have difficulty answering any of the five questions? (If yes, which one and why?)

__________________________________________________________________________

___________________________________________________________________________

c. Did you have any difficulty completing the VAS? (if yes, what was the difficulty?)

__________________________________________________________________________

___________________________________________________________________________

2. Did you find completing the electronic EQ-5D-5L questionnaire before the doctor consultation useful?

If yes:

a. How was it useful? __________________________________________________________

__________________________________________________________________________

___________________________________________________________________________

b. Which part was useful and why?

__________________________________________________________________________

___________________________________________________________________________

3. Would you be willing to complete the electronic EQ-5D-5L questionnaire before the doctor consultation regularly?

If Yes, why? _______________________________________________________________

__________________________________________________________________________

If No, why? _________________________________________________________________

**Appendix 6: Characteristics of Subjects and Key Quotations by Themes of Qualitative Study**

**Supplementary Table 1: Characteristics of Subjects of Qualitative Study**

| **Characteristics of Doctor Focus Group Participants** | | | | |
| --- | --- | --- | --- | --- |
| Code | Sex | Age | Years of Experience as a doctor | Number of times the participant had reviewed the e-EQ5D5L reports* |
| G2, Dr. A | M | 46 | 22 | 89(T0:39/ T1:28/ T2:22) |
| G2, Dr. B | F | 44 | 19 | 20(T0:15/ T1:2/ T2:3) |
| G2, Dr. C | M | 44 | 20 | 55(T0:23/ T1:16/ T2:16) |
| G1, Dr. D | F | 45 | 20 | 56(T0:23/ T1:17/ T2:16) |
| G1, Dr. E | F | 42 | 18 | 60(T0:29/ T1:21/ T2:10) |

| **Characteristics of Patient Feedback Interview Participants** | | | | | | | | | |
| --- | --- | --- | --- | --- | --- | --- | --- | --- | --- |
| Code | | Sex | Age | | Education level | Occupation | | Number of chronic disease(s) | Number of times that they have filled out the e-EQ5D5L* |
| ALC_PT1 | | F | 69 | | Primary or less | Labour work | | 2 | 3 |
| ALC_PT2 | | F | 69 | | Primary or less | Labour work | | 1 | 3 |
| ALC_PT3 | | F | 63 | | Secondary | Clerical work | | 2 | 3 |
| ALC_PT4 | | M | 61 | | Secondary | Labour work | | 2 | 3 |
| ALC_PT5 | | F | 67 | | Primary or less | Retired | | 1 | 3 |
| LT_PT1 | | F | 73 | | Primary or less | Retired | | 1 | 2 |
| LT_PT2 | | M | 65 | | Primary or less | Retired | | 3 | 3 |
| LT_PT3 | | M | 60 | | Secondary | Labour work | | 1 | 3 |
| LT_PT4 | | M | 57 | | Secondary | Labour work | | 3 | 3 |
| LT_PT5 | | M | 40 | | Secondary | Labour work | | 3 | 2 |
| UCH_PT1 | | F | 71 | | Primary or less | Homemaker | | 2 | 3 |
| UCH_PT2 | | M | 79 | | Primary or less | Retired | | 1 | 3 |
| UCH_PT3 | | M | 82 | | Primary or less | Homemaker | | 1 | 3 |
| UCH_PT4 | | F | 50 | | Primary or less | Homemaker | | 1 | 3 |
| UCH_PT5 | | F | 64 | | Primary or less | Homemaker | | 5 | 3 |
| **Characteristics of Research Assistant Focus Group Participants** | | | | | | | | | |
| Code | Sex | | | Age | Number of months involved in the study | | Number of times that they had assisted the data collection with the e-EQ5D5L * | | |
| RA1 | F | | | 27 | 13 | | 380(T0:79/ T1:170/ T2:131) | | |
| RA2 | M | | | 23 | 17 | | 669(T0:308/ T1:220/ T2:141) | | |
| RA3 | F | | | 26 | 7 | | 17(T0:0/ T1:2/ T2:15) | | |

Notes

*Cut-off date for all info: 31-Dec-2021; T0 = baseline, T1= 1^st^ follow up, T2 = 2^nd^ follow up, M = Male, F = Female

**Supplementary Table 2: Themes and Subthemes on Patients’** **PEOU and PU with Quotations from Patient Interviews and RA Focus Groups**

| **Themes** | **Subthemes** | **Quotations** |
| --- | --- | --- |
| **Perceived ease of use (PEOU) in terms of methods of administration** | | |
| Difficulty in using an e-platform | Technology-related problems | [I] do not know how to use electronics. [I] wait for the youngster to use and read it out for me. [LT_PT3]  Some of them did not even know how to use electronic devices, so they gave up using [the iPad] and asked us to operate [for them] instead. [RA2] |
| Difficulty of self-administration | Vision-related problems | Some words are too small. [It’s] hard to see it clearly. It is better to have someone reading out the questions. [LT_PT1]  As some elderlies might have presbyopia and they would need to take their glasses out in a rush. They might not be able to view the iPad clearly. [RA1] |
|  | Requiring assistance to understand the survey question | It is clearer if someone reads it out to me. [ALC_PT1]  In terms of the EQ-5D questionnaire, we need to explain the questions [to the patients]. They cannot answer clearly. We will lead them slowly and try to explain all the options [of each question] to them so that they could answer the questions… They actually need our help to finish those questions. [RA2] |
|  | Literacy problems | [I] could do it, but could not do it by myself. It is possible for you to find someone to explain it to me. I am not literate and afraid that I will do it wrongly. [ALC_PT5] |
|  | Increasing age | Usually, patient did not know how to answer the questions, so we need to explain [them to patient]. Especially for the elderly, we need to explain. I have experienced that some of the younger people might grab our iPad to answer the questionnaire [without our help]. Therefore, age is a major factor affecting the completion [understanding] of the questionnaire. [RA2] |
| **Perceived ease of use (PEOU) in terms of questions** | | |
| Difficulties in understanding | Unclear definition of the terms | Yes, I am not clear about how to answer the questions. [It is] not stated clearly. [ALC_ PT5]  For “mobility’, maybe they could not differentiate if it referred to normal walking or going up and down the stairs. Some of them might say “No problem with normal walking, but it was hard to go up and down the stairs”. [RA2]  Some questions ask about “to what extent to do something” and “to agree or disagree” with it. And I think the elderly may not understand the magnitude of “to what extent” in the questionnaire. [RA3] |
|  | Improved understanding after repeated use | [I] probably know what will be asked after answering for a few times. [LT_PT4]  For the younger generation of around 50- to 60-year old, they already knew what our questions are after answering for a few times. They might remember and answer [the questions by themselves] even before we read the questions, they are able to respond. For the older ones, it took us more time to explain the questions in order to answer what we were asking. But for most of them, after doing it for several times, it would be easier to ask them compared with the baseline. [RA1] |
| Difficulties in answering | Unable to describe own health in levels | Classification [of levels] is probably okay, but I am not sure whether I classified [the levels] correctly or not. It is hard to understand the difference. [ALC_ PT5]  Most of the patients said that they do not know which option to choose or they don’t really know their level, then they will ask us to choose randomly. They may not provide an answer that we expect. So in these situations, it is harder to pick. [RA1] |
|  | Unable to specify score due to fluctuating health conditions | It is not that difficult but I need to think about how to answer. I feel painful sometimes and sometimes do not. Does it count as absent or mild? I do not know how to classify. [UCH_PT4]  [The patients] would say “from 0-100, it is really hard to answer. For example, I felt great pain in the morning, but then I took 2 tablets of analgesics, then I feel better now. Or else I could not come to see the doctor now. So, I don’t know what score to give myself”. [RA1] |
|  | Providing a score range instead of an exact score | With respect to taking (VAS) score, most of the patients can make the decision. From 0-100 they may not give me a precise number, but they can give me the range of the score (e.g. score 50-60) by themselves. [RA1] |
|  | Perceiving the response options in e-EQ-5D-5L/VAS as too “severe” | Patient thought it was hard to walk due to the pain, but he/she also thought if he/she chose “severe” then it would really seem to be exaggerating. Then he/she said “how about I choose ‘mild’ instead” because he/she thought if he/she chose the term “Severe”, it would mean that he/she was having a serious problem. [RA3] |
| **Perceived usefulness (PU)** | | |
| Usefulness to patients | Understand the patient situation | Yes, let the doctor to review the situation. The doctor will discuss with me. [ALC_ PT3] |
|  | Helpful for treatment | At least the doctor knows whether I feel painful or not. So he will prescribe medicine. [LT_PT2] |
|  | Uncertain usefulness | I do not know [if it is useful]. It should be. [But] I do not understand [it]. [UCH_PT4] |
|  | Not useful to patient | [Usefulness] depends on the doctor. The doctors here could not help me. [They] only help by writing a referral to orthopaedics. [UCH_PT2]  I think it is useless. You can figure it out by statistic. We do not have much feeling. [UCH_PT3]  Usually, if they were on analgesics, then they got prescribed analgesics again. There was no other [treatment given by the doctors]. The doctor might think that the situation of the patient was not severe enough for an orthopedic referral. The patient may have wanted to go [to see orthopedic] but the doctor did not think there was a need. Therefore, the patient might think [that the report] is not useful. [RA1]  Previously, one of the patients said that “the interview was not useful as the doctor would not see the report”. [RA3] |
| Usefulness to others | Useful to researcher | It's just useful to you. I take it as chatting with you. When I see the doctor, he would not offer any extra medication or say anything. [ALC_ PT1]  And also, it helps for the research purpose. [I’m] just waiting for seeing the doctor anyway. [LT_PT3] |
|  | Useful to other patients | I think that doing this survey could help the others. [UCH_PT3]  It also helps for the research purpose. [I’m] just waiting for seeing the doctor anyway. [LT_PT3] |
| **Feasibility** | | |
| Time for completing the e-EQ-5D-5L/VAS | Short completion time | [It takes] a few minutes to answer. There should be no problem to do it when waiting [for consultation]. [ALC_ PT2]  Usually, I will interview them quickly by asking them the question and choices, which actually takes within 5 minutes at all. [RA2] |
|  | Feel like chatting | [I] answer quickly and easily when someone is reading it out. It’s like chatting. [LT_PT3] |
|  | Difficulties with finding the patients in the clinic | The major issues are when the patient arrives, and how fast we can locate the patients. [RA1]  There were some difficulties in locating the patients in the clinic because they had to go to measure blood pressure or go to the restroom. It might be hard to find the patient. The patients might arrive very late, so I could not be sure whether they had attended the clinic or not. [RA2] |
|  | Slight impact by unstable network | Sometimes when the overall reception was not that good, it would get disconnected. It would take a longer loading time when connecting to the e-platform, but overall, it did not add too much time. If a connection issue occurs, I might need to wait longer to export and print the [e-EQ5D5L] report. [RA1] |
|  | Limited time before consultation | It depends on the time when the patients arrive [at the clinic]… If they arrive with not much time left before their consultation, then they need to rush to measure the blood pressure and also to see the doctor, then the whole logistics will be busier and more rushed. They may even say that we are impeding them to see the doctor, so this time they don’t want to do it. Then just rush in to see the doctor and forget it. [RA1] |
| Time for consultation | Time-saving by knowing their painful condition before the consultation | It is useful. The report given to the doctor directly simplifies the process. The doctor just has a look and knows. He does not need to ask again. It is convenient for [the doctor and patient]. [UCH_PT5] |
| Poor patient attitudes | Annoyance by repeated surveys | Patients felt annoyed during the follow-up interviews because they have done it for around 3 to 4 times. They will make comments like “need to do it again?” [RA2]  The patients were fine and willing to help us do the interview the first 1-2 times. However, starting from the 3rd to 4th time, patients might ask “why is the study not finished?”, “every single time I come back, you always find me but doctors only give analgesic to me and do not have any other help.” [RA1] |
|  | Perceiving (the e-EQ-5D-5L/VAS information) useless | That patient may think that the doctor kept the same treatment after viewing the report and s/he did not want us to keep asking them question, so that patient chose to answer “No pain”, “no problem”, “you don’t need to look for me” and quickly finished it. At the end, s/he gave the report to the doctor who reduced the medication. [RA1]  The patient said that every time the doctor doesn’t look at it, so he does not want to do it. [RA3] |

**Supplementary Table 3: Themes and Subthemes Doctors’ PEOU and PU with Quotations from Doctor Focus Groups**

| **Themes** | **Subthemes** | **Quotations** |
| --- | --- | --- |
| **Perceived Ease of use (PEOU) in terms of viewing the report** | | |
| Clarity of information | Clear layout of the report | Well, it's actually quite clear to see. [G2, Dr. A] Yeah, all right it's quite clear. [There] is the figures. There is a graph and it's easier to see it. [G2, Dr. C] |
| Ease of interpretation of report | Easy to compare with the population mean | I can compare it to the Hong Kong population median so to see the patient's disease. [Like] how severe [it] can affect the patient when it [is] compared to the general population." [G2, Dr. C] |
|  | Easy to see the trend of scores | I can know the exact point and the trend of the point, whether it is increasing or decreasing, and it is very easily understandable. [G1, Dr. D] We can see this chart and see that the patient is deteriorat[ing] or already improv[ing]. [G2, Dr. B] |
| Limitations in interpretation | Other confounders of the representation | I'm not sure whether patients… are asked to assess the score just for their musculoskeletal problems or as a whole picture. Because for our patients, many have multiple medical problems. So, I think sometimes it may not just reflect [the MSK problem] if there are a lot of confounders… Because he has limited activities or poor sleep, not just because of the pain or the musculoskeletal skeletal problems, or maybe it is just related to congestive heart failure or other problems. It is more complicated in our clinic, I think. [G1, Dr. D] |
| **Positive feelings towards the perceived usefulness (PU)** | | |
| Understand the patient better | Better understanding on impact of patient's daily living | The EQ5D score will give us a more comprehensive assessment of the patients on the condition, especially on how the disease affects his or her daily life. [G1, Dr. D]  I think the overall [score] is helpful and it's stimulating because it stimulates how I should pay attention to the other parts of patient, particularly the psychological part. [G2, Dr. A] |
|  | Monitoring the progress of MSK condition | I agree that if the patient mainly comes for MSK issues, this is good tool to monitor the progress of the condition. Just like in other clinics for the mental health, the PHQ or GAD score is [a] good reference for monitor[ing] the progress of the disease. [G1, Dr. E] |
|  | Useful for less active patients or patients with MSK issues as the chief complaint | But if the chart comes, I read the chart first and found that actually, the score is actually quite alarming, then I would actively address the problem and then ask the patient on that. It is because the patient actually doesn't mention about the pain, but actually the pain is not improving… so I really need to address them. [G2, Dr. A]  If the patient is to follow up for the musculoskeletal pain problem, I think this chart is very useful. [G2, Dr. B] |
|  | Prompting a discrepancy in pain perception between doctor and patient | If the score is lower than I expected… I think it is very useful because what I perceived may not be the same as the patient. That made me understand the patient more, how does it affect him or her, so I think it may be very useful when I encounter this kind of patient. [G2, Dr. A] |
| Manage the patient better | Increased lifestyle management/counseling patients | ... but I will talk a little bit more to them. Because I think sometimes not only active intervention can change the disease progress. Sharing and counseling is also an important part. Actually, active intervention, I don't have much. I cannot do much. Maybe spend one minute or more time to talk to them. [G1, Dr. D] |
|  | Selecting treatment based on the trend | ... another point is that we can look for the treatment. Because we usually give a few different treatments, [such as] advice on the lifestyle and also medications okay, and also may refer to the Physio. But… the score… can actually stimulate us to think more specifically, [about] which particular treatment would help. Say, for example, some people will be actually having the same body weight; but actually, the pain is getting much better so [it] is very likely to be some physical therapy effect. [G2, Dr. A] |
| **Negative feelings towards the perceived usefulness (PU)** | | |
| Situations where report is not useful | Patients who already actively share about their MSK problems during consultation | Actually, our patients are very willing to tell us their musculoskeletal problems during follow-up and even [if] they are not being followed-up because of the musculoskeletal problems. [G1, Dr. D]  And I think, if the patients…musculoskeletal problem [is] bothering them, they will volunteer themselves. [G1, Dr. E] |
|  | (Patient) having follow-up by a specialist for MSK problem/ not coming for MSK issues | Usually, we do not have a lot of modification for the management because sometimes the patients having musculoskeletal problems in our clinic may have proper follow-up by the orthopedic clinic and they already provide other intervention, so we usually do not have any extra intervention such as the physical therapy or other intervention for the patient. [G1, Dr. E]  If the patient didn't complain about the problem, I don't think the report is useful. Because he or she [is] already followed by the specialist and I think they will tackle the problem by themselves. Okay, so if the patient actually [isn’t] bothered by that during the consultation by our GOPC, I think that score may not be helpful. [G2, Dr. B] |
| Aspects of care not addressed | Patient needs are better communicated verbally than by a score | Because I understand the patient’s need is very variable and it cannot just be reflected in the score even it is very low … That’s because - “the needs” - that we have to ask exactly what the patient actually needs but cannot just be reflected by the score. [G1, Dr. D]  For me, “the needs” means some expectation at the back of [the] patient’s mind. So sometimes we need to go into the consultation to get the need rather than just looking at the figures. [G2, Dr. A] |
| **Feasibility** | | |
| Time for interpretation | Quick reference | It is not time-consuming. It's very easy to understand, and I just have a look at the score, and I can know that. [G1, Dr. D]  Right! It costs me just about one minute. [G2, Dr. A] |
|  | Time-saving by knowing the patient’s pain condition before the consultation | If [the score] is similar as before…I think the patient will not tell me about the pain so it's helped me to reduce my consultation time actually because I already know that he or she is suffer[ing] from the pain or not." [G2, Dr. B] |
| Time to address the result | Balancing between the usefulness and additional time for addressing the MSK problem | It is always helpful to have all these scores…But it also costs me. Although the time of reading and analyzing is not too much, sometimes we need time. Because of that, we need to think a little bit more and that's using a little bit more time. Even if I need to treat their mood problem, then I will use that even more time. [G2, Dr. A] |
|  | Limited consultation time/ The need in addressing other medical problems (in the same consultation) | After understanding the score, I think sometimes we don't have enough time to deal with it... because, apart from the joint or musculoskeletal problems, many patients have different other medical problems and to deal with. [G1, Dr. D]  We really have time constraint during our consultation. We need to manage more than one to two problems and sometimes three or four problems. Although [it takes] less than one minute, [it] may also [be] very stressful for the doctors. [G2, Dr. B] |
